# Still invisible? Legal and ethical treatment of intersex individuals in China

**DOI:** 10.1093/jlb/lsag018

**Published:** 2026-06-04

**Authors:** Meng Wang, Yann Joly

**Affiliations:** Faculty of Law, University of Macao, 999078; Centre of Genomics and Policy, Victor Phillip Dahdaleh Institute of Genomic Medicine, McGill University, H3A 0G1

**Keywords:** intersex, comparative law, human rights

## Abstract

This article explores the legal, social, and ethical challenges faced by intersex individuals in China, a population estimated to number in the millions but still largely invisible in national laws and public discourse. Drawing on recent international human rights developments and comparative legal analysis, the paper critically examines China’s legal and medical frameworks through the lens of intersex rights. It highlights how entrenched binary conceptions of sex and gender deeply rooted in Confucian traditions, and reflected in Chinese administrative, medical, and legal systems, lead to widespread discrimination, non-consensual medical interventions, and structural exclusion of intersex individuals. The authors argue that despite generic constitutional and civil guarantees of equality, bodily integrity, and informed consent, intersex persons remain insufficiently protected due to the absence of targeted legislation and interpretive guidance. The article proposes incremental yet concrete reforms such as deferring non-urgent medical interventions, improving psychosocial support, introducing neutral terminology, and developing best-practice medical guidelines as viable steps toward greater inclusion. By examining relevant international and regional practices, including developments in Malta, Australia, and Hong Kong, the paper advocates for a post-binary legal approach that affirms intersex individuals’ dignity, autonomy, and right to recognition in Chinese law and society.

## I. CONTEXT

With the rapid advancement of technology, the advocacy of human rights organizations, and the gradual enhancement of social awareness, discussions surrounding sex and gender diversity are receiving growing attention on a global scale.^1^ In particular, the intersex community, which was previously ‘invisible’ to most, has now gained increasing attention in international discourse.^2^

A recent report from the Office of the United Nations High Commissioner for Human Rights (OHCHR) describes intersex people as: ‘individuals born with physical sex characteristics—such as chromosomes, gonads, hormone levels, or reproductive anatomy—that do not fit neatly within the strict binary understanding of sex (i.e., male or female classifications).’^3^ A 2016 survey highlights over 40 recognized intersex variations globally.^4^ These variations can be identified at different stages of life, with some manifesting early, such as Partial Androgen Insensitivity Syndrome (PAIS), and others emerging later, like Complete Androgen Insensitivity Syndrome (CAIS). Certain variations, like Congenital Adrenal Hyperplasia (CAH), may require immediate medical intervention due to their life-threatening nature, while others, such as Klinefelter Syndrome, may go undiagnosed for years due to subtle symptoms.^5^

While a variety of terms, such as ‘disorders of sexual development’ (DSD), ‘differences (or variations) of sexual development’, and ‘diverse sex characteristics’, are currently used in medical and public discourse, advocacy groups like interACT have increasingly favored the term ‘intersex’ in their communications.^6^ It is also the term preferred by Chinese intersex advocates, and is commonly translated into Chinese characters as ‘双性⼈’ or ‘间性人.’^7^ Although ‘DSD’ remains common in clinical settings, many individuals with intersex traits reject it due to its pathologizing connotations.^8^ In this article, we adopt the term ‘intersex’ to align with the terminology promoted by the international intersex human rights movement and Chinese intersex advocates.^9^ We acknowledge, however, that terminology in this field continues to evolve, and preferences may vary across contexts and individuals.

Despite intersex traits representing a naturally occurring aspect of human and sex diversity, global attention and legislative protections for this community remain insufficient.^10^ Significant disparities persist among countries regarding the adequacy and effectiveness of protective measures, with diverse ethical challenges and legal protections for intersex individuals identified across different jurisdictions.^11^

In China, while sex and gender diversity are acknowledged within academic circles, intersex individuals face particular challenges within society, the healthcare system, and legal frameworks.^12^ Although some initial progress has been made in the Hong Kong Special Administrative Region,^13^ including obtaining some recognition from policymakers within the government and growing support from civil society organizations,^14^ such progress remains confined to this region. These changes in Hong Kong have yet to significantly influence public perception or governance practices in mainland China. Research in this country continues to indicate widespread misunderstandings and stigma regarding intersex individuals,^15^ with many families reluctant to acknowledge any variations in their children’s sex characteristics that diverge from traditional binary norms.^16^

Furthermore, in past court cases involving intersex individuals in China, their biological sex was often not properly recognized, particularly in cases of sexual violence and human trafficking.^17^ At the time, the legal framework recognized only women as victims of these crimes. As a result, intersex victims were frequently misclassified as female, and their biological characteristics or gender identities were omitted from judicial decisions.^18^

This lack of understanding, compounded by societal stigma, results in the marginalization and potential discrimination of intersex individuals. Notably, the intersex population in China is estimated to be substantial. Despite considerable variations in definitions and scope used to estimate intersex populations, authoritative estimates suggest that approximately 0.05 to 1.7 percent^19^ of the global population is intersex.^20^ Applying these estimates to China indicates that there could be between 7 million and 23.8 million intersex individuals nationwide. These numbers underscore the urgent need to address gaps in societal recognition, healthcare inclusion, and legal protections of the intersex community in China.

These challenges may stem largely from China’s sociocultural norms, influenced by traditional Confucian beliefs that emphasize clearly differentiated social roles for men and women,^21^ and establish expectations regarding reproduction and the continuation of family.^22^ These beliefs assign distinct reproductive roles exclusively to men and women. Consequently, they reinforce rigid conceptions of sex and gender, which render invisible those who do not fit binary categories.

The strong cultural emphasis on maintaining tradition has contributed to the endurance of these norms.^23^ Rather than being questioned or re-evaluated, this traditional understanding is often preserved as a moral ideal to be upheld across generations. As a result, binary conceptions of male and female bodies remain the mainstream understanding in contemporary Chinese society.^24^ This cultural context places considerable social pressure on families of intersex children, leading to fears that their child’s variation may expose them to various forms of social exclusion or discrimination, ranging from subtle stigma to overt hostility and rejection.^25^ Consequently, many intersex children may undergo ‘gender normalization’ surgeries shortly after birth to align their bodies with prevailing binary standards.^26^ These procedures are often performed without obtaining informed consent from the children themselves, as decisions are frequently made by parents and medical professionals based on societal expectations, rather than around children’s best interests, autonomy, and psychological well-being.^27^

The persistence of rigid sex and gender categorizations is reinforced by legal and administrative practices that officially recognize only two genders. Such institutional frameworks, combined with traditional cultural beliefs, create additional obstacles for intersex individuals.^28^ Additionally, under China’s legal system, judicial decisions are not bound by precedent. Judges retain discretion to decide each case with care and based on its particular facts. As a result, legal protections for the intersex community may vary from case to case, leading to inconsistent outcomes.

In summary, there remains a considerable need in China to enhance public understanding and social acceptance of intersex individuals. Going forward, it will be essential for Chinese scientists, ethicists, and policymakers to unambiguously affirm that intersex people are a natural part of human diversity and should be welcomed as full members of society. While global attention to the rights of intersex individuals is gradually increasing, this trend has yet to fully reach China, where the legal framework protecting intersex people has not yet been critically appraised. With these challenges in mind, this article first seeks to identify the socio-ethical and cultural issues that have contributed to shaping the treatment of intersex people under Chinese law. It then examines how Chinese law and medical governance currently regulate the treatment of intersex persons and identifies key gaps in protection relating to bodily integrity, informed consent, and equality. It argues that existing rules, spread across civil law, administrative regulation, and clinical practice, create a largely medicalized and paternalistic framework. Drawing on comparative examples from selected jurisdictions, the article proposes incremental legal and policy reforms that could strengthen protection for intersex persons in China.

## II. ETHICAL CHALLENGES FACED BY INTERSEX INDIVIDUALS IN CHINA

### II.A. Social Context: Marginalization and Discrimination

#### 1. Invisibility and Persistent Misunderstanding

Though Confucian philosophy has had a lasting influence on Chinese culture, particularly as it relates to the representation of a strict sex and gender binary,^29^ there do exist some ancient texts that document the physical characteristics of intersex individuals.^30^ Still, these texts were largely intended for medical professionals and were not used to familiarize the broader population with the existence of the intersex community.^31^ In contrast, publicly accessible works, such as ancient literature, mythological stories, and popular novels, often depicted intersex individuals as either mysterious figures or marginalized, anomalous beings.^32^ This portrayal has reinforced societal stereotypes and deepened misunderstandings about intersex individuals in China over time.

Today, although modern Chinese society’s views on sex and gender have progressed, partly driven by advances in advocacy, science, and medicine, public awareness of intersex people remains limited.^33^ A binary understanding of sex and gender continues to shape many aspects of everyday life, including the gendered design of public facilities. Most public restrooms are still designed exclusively for male and female users, with little consideration given to the needs of intersex, transgender, and gender-diverse individuals.^34^ Although gender-neutral restrooms have emerged in some areas in recent years, their adoption remains limited and is largely confined to specific spaces, such as high-end commercial venues and tourist attractions.^35^ In most public spaces, restrooms continue to operate on a strictly binary framework, causing some intersex individuals to face discomfort or stigmatization when using these facilities.^36^

In public discussions, it has been suggested that sex and gender minorities, including intersex individuals, should ‘choose restrooms based on their appearance’ to avoid confusion or controversy.^37^ This approach assumes that an individual’s external appearance always aligns with their gender identity and sex classification, which is not the case for all intersex individuals. It fails to respect intersex individuals’ autonomy to choose their gender identity, reinforcing the very binary framework that excludes them in the first place, rather than offering a meaningful solution. Finally, it overlooks intersex-specific challenges such as intersex person’s sex characteristics not necessarily aligning with what is expected in a given restroom.

Similarly, the binary classification system is omnipresent in healthcare access and administrative processes. Many Chinese medical institutions lack inclusive policies, expertise, and appropriate facilities for intersex individuals.^38^ For example, patients are often asked to select either ‘male’ or ‘female’ during registration or triage, without an option to list having an intersex variation, which prevents intersex-specific care and excludes those whose identities fall outside the binary framework.^39^ Additionally, many healthcare professionals may lack adequate training or awareness regarding the unique needs of intersex individuals, which can inadvertently cause psychological harm.^40^ For instance, some intersex patients report being repeatedly asked to explain their variation during consultations, significantly exacerbating the stress and discomfort they associate with the healthcare system.^41^ This lack of sensitivity and preparedness not only hinders access to equitable healthcare but also reinforces societal denial of their identities.

Meanwhile, mainstream societal perceptions and media portrayals of intersex individuals remain steeped in bias and misunderstanding. In public discourse, intersex individuals are often viewed as ‘abnormal’ or as deviating from societal or familial expectations.^42^ Even reports intended to promote understanding and acceptance frequently frame intersex individuals as ‘patients’ and emphasize the necessity of medical interventions to ‘restore normalcy’ in line with societal expectations.^43^ Media reports often describe medical procedures on intersex children as ‘corrective surgeries,’ positioning them as necessary for helping the child to ‘develop a normal personality.’^44^ Although these media reports have provided some visibility to the intersex community, their misrepresentation of intersex identities and needs solidifies and perpetuates the public’s misperception of intersex variations as pathological and deviating from the norm.

#### 2. Employment Discrimination: Barriers to Social Integration

Employment discrimination remains a significant barrier to the social integration of intersex individuals, hindering their ability to secure stable livelihoods and participate fully in society. Past research has consistently shown that, beyond the typical challenges faced by all jobseekers, intersex individuals encounter unique biases, which are often rooted in societal attitudes toward sex and gender diversity and nonconforming sex characteristics.^45^

In China, it is still commonplace for recruitment processes to require candidates to submit detailed health assessment reports.^46^ For intersex individuals, this practice presents particular challenges, as such reports may inadvertently disclose sensitive medical histories or reveal their intersex status. This disclosure can expose individuals to discrimination, significantly diminishing their job prospects and social integration.

A landmark case in China involved the dismissal of a gender-nonconforming individual (Mr C), whose registered identity and health assessment report indicated female, but who identified and presented as male, exemplifying such discriminatory practices.^47^ Despite the health assessment report showing no health issues, the employer cited ‘unhealthiness’ and ‘impact on the company’s image’ as reasons for the dismissal.^48^ The dispute first entered the labor arbitration stage, which is a mandatory preliminary procedure for most labor disputes in China.^49^ The arbitration decision supported Mr C’s requests for unpaid wages and compensation for the unlawful termination of the employment contract but did not address the issue of gender discrimination.^50^ Mr C subsequently filed a lawsuit against the employer, alleging a violation of the personality rights, seeking to challenge the broader pattern of discrimination faced by individuals with similar gender identities in the employment environment. The court later held that the employer had violated Mr C’s right to equal employment by terminating the employment contract without reasonable justification and awarded RMB 2000 (approximately USD 280) in compensation for emotional distress.^51^ However, although the court acknowledged in its judgment that an individual’s gender identity and gender expression should be protected and respected in accordance with the law, it declined to recognize the employer’s conduct as constituting gender discrimination on the grounds that the evidence presented was insufficient.^52^ This case likely illustrates a common bias against individuals who do not conform to traditional sex and gender norms perpetuated in China’s current employment environment.

A 2020 survey conducted in China further revealed that intersex individuals who had undergone early gender-assignment or so-called ‘normalizing’ surgeries were particularly vulnerable to discrimination in the workplace.^53^ In many cases, discrepancies between their identification documents, medical records, and gender identity often led to job rejections, placing them in precarious employment situations and contributing to unstable living conditions.^54^

The lack of stable employment may place intersex individuals at a higher risk of poverty, which in turn restricts their access to essential resources like housing, healthcare, and education. This exclusion reinforces their social marginalization and poses a broader ethical challenge undermining the principles of fairness and social justice by denying individuals the right to participate fully in society. It also points to the intersectionality of discrimination, which impacts these individuals not only for reasons of sex and gender non-conformity but also contributes to furthering their marginalization, which can expose them to other types of discrimination later in time (for example, based on economic condition, education level, disability, etc.).^55^

### II.B. Medical Contexts: Non-Consensual Interventions and Therapeutic Privilege

Another significant ethical challenge faced by intersex individuals in China arises in the medical context, particularly with regard to non-consensual interventions and therapeutic privilege. Past cases in China have shown that interventions for intersex individuals, including surgeries and hormone treatments, are often carried out at a very young age, before the individuals are capable of providing consent.^56^ Some of these interventions are performed to address serious and immediate health risks, such as conditions that threaten the child’s life or impair vital bodily functions, and are generally regarded as both medically necessary and ethically permissible when undertaken early. In contrast, other procedures, particularly so-called ‘normalizing’ surgeries, are carried out solely to align a child’s body with binary sex and gender norms.^57^ These lack urgent medical justification and raise more serious ethical concerns, especially when performed before the intersex individual is mature enough to meaningfully participate in the decision-making process. Decisions regarding treatment are also typically made jointly by parents and doctors,^58^ raising ethical concerns about autonomy, bodily integrity, and the right of intersex individuals to make fully informed decisions about their own medical care.

More generally, the lack of informed consent in medical decisions involving children remains a persistent ethical problem in China.^59^ Although informed consent is a cornerstone of medical ethics, ensuring that individuals can make decisions based on a comprehensive understanding of treatment risks, benefits, and potential consequences, it is impossible for infants and young children to provide such consent.^60^ In some jurisdictions, such as Greece,^61^ Malta,^62^ and Spain,^63^ alternative mechanisms have been adopted, particularly for medical decisions that significantly affect a child’s bodily integrity and future identity, such as those involving intersex traits. In such cases, non-urgent procedures are often delayed until the child is mature enough to give fully informed consent, or assent is sought when they reach an appropriate age.

In contrast, China has not yet fully developed comparable mechanisms. China’s medical regulations and guidelines for children’s healthcare primarily emphasize obtaining parental informed consent, often overlooking the child’s developing autonomy and consent rights.^64^ As legal guardians, parents are entrusted with the authority to make medical decisions on behalf of their children.^65^ Although Article 35 of China’s Civil Code requires guardians to act in the best interests of the ward and, in the case of minors, to respect their genuine wishes in accordance with their age and level of understanding, this principle is not always consistently implemented in medical contexts. Obtaining assent from children for medical procedures and postponing non-essential interventions to allow for their informed consent at a later stage are not common practices in China.

Intersex medical care in China largely follows a ‘paternalistic’ model, where parents and doctors, rather than the children themselves, make crucial treatment decisions invoking the welfare of the child or following on strongly embedded social norms.^66^ Many parents, influenced by social stigma and traditional views on sex and gender, worry that their child might face discrimination or marginalization in the future. Consequently, they often agree to early so-called ‘gender-normalizing’ procedures, hoping this will shield their child from future social exclusion.^67^ Additionally, due to a lack of sufficient medical knowledge, parents tend to rely heavily on the expertise of doctors, who often play a dominant role in determining when and how such treatments proceed.^68^ In such cases, the medical community in China often perpetuates practices historically observed in other countries,^69^ focusing on conformity to binary sex and gender expectations and advocating for early medical interventions.^70^ The interaction of societal pressure and medical authority leads parents to often disregard the will and autonomy of their intersex child.

This medical model often misses the important differences in urgency, medical justification, and long-term consequences across the range of procedures commonly performed on intersex children. Typically, as in many other countries, medical interventions for intersex children in China fall into three categories:^71^ (i) Surgery to address serious and urgent health conditions (eg life-threatening complications such as urinary obstruction or Salt-wasting CAH that pose immediate risks); (ii) Surgery for non-urgent but potentially health-related concerns (eg removal of streak gonads or undescended gonads due to cancer risks); and (iii) Cosmetic surgery only intended to make the child’s body conform to binary sex and gender norms (eg genital reconstruction conducted for social or esthetic reasons rather than medical necessity). While the first category is generally considered ethically acceptable with physician recommendation and parental consent, the second and third raise significant ethical issues due to their non-urgent nature. In many jurisdictions, such as Greece, Malta, and Spain mentioned above, best practices recommend deferring non-urgent or cosmetic procedures until the child is mature enough to participate in decision-making and provide informed consent.

Despite these distinctions, China’s current medical framework regarding intersex traits does not always clearly differentiate between urgent and non-urgent procedures. In practice, non-urgent medical interventions, particularly those driven by binary sex and gender norms, remain common.^72^ While there are some opinions in Chinese medical literature suggesting that such procedures should be postponed until the child can give informed consent,^73^ these perspectives remain in the minority.

This paternalistic decision-making model also has profound implications for intersex individuals later in life, especially regarding long-term physical and psychological harm. Physically, surgeries such as genital reconstruction or gonadectomy, commonly performed in infancy or early childhood, can lead to a range of permanent consequences.^74^ These may include loss of sexual sensation, chronic genital pain, scarring, and, in many cases, irreversible infertility.^75^ In adulthood, some intersex individuals may also require lifelong hormone replacement therapy or undergo additional corrective surgeries, which can impose lasting medical burdens and impact their overall health.^76^ On a psychological level, many intersex individuals experience lasting emotional distress and struggles with their identity due to irreversible bodily changes.^77^

Moreover, another critical ethical issue in the context of medical decision-making for intersex individuals in China relates to the practice of withholding information under what is known internationally as therapeutic privilege. This ethical and legal principle allows healthcare professionals to withhold diagnostic results or limit disclosure if the full information is deemed potentially harmful to the patient’s well-being or not in their perceived best interest.^78^ In intersex care, such withholding may prevent parents from fully understanding their children’s intersex traits and the long-term consequences of early treatments, especially as they relate to irreversible surgeries performed on intersex infants.

In China, the term therapeutic privilege itself is not formally recognized in legal or ethical guidelines. A functionally similar concept to what is known in Western countries as therapeutic privilege referred to in China as ‘protective medical measures’ exists in medical regulations and clinical practice. It serves a comparable purpose, allowing doctors to withhold or limit certain information when disclosure is judged to be potentially distressing.^79^ To facilitate understanding among international readers, this article uses the internationally recognized term therapeutic privilege throughout when referring to this practice in the Chinese context.

Although Chinese medical law formally incorporates the principle of informed consent, empirical and legal scholarship suggests that disclosure practices often remain mediated by families and clinicians in China, who may decide to withhold information from patients in order to protect them from psychological harm.^80^ This practice has been described as reflecting a family-oriented model of consent that can function similarly to the doctrine of therapeutic privilege. While direct empirical evidence regarding its application in intersex care in China remains limited, such patterns in clinical communication suggest that comparable practices may arise in this context. For instance, a recent survey of hospital doctors in China found that over half of the respondents doubted patients’ capacity to make rational decisions or believed that patient involvement in treatment would not improve outcomes.^81^ Such beliefs, coupled with the perceived complexity and sensitivity of disclosing an intersex variation, likely exacerbate physicians’ concerns about parents’ capacity to make informed decisions in this context. Even when there is a partial or complete disclosure, parents, lacking specialized knowledge about intersex variations, often rely entirely on the judgment of medical professionals, further amplifying the effects of this paternalistic model.

An additional factor influencing intersex-related medical decision-making in China is the language and framework used to describe intersex individuals. The Chinese medical community largely adheres to the 2006 Chicago Consensus, which classifies intersex variations as ‘DSD.’^82^ While this classification aims to standardize care, it inadvertently pathologizes intersex traits, reinforcing the perception of these variations as medical abnormalities requiring correction. This pathologizing language, combined with the authority of medical professionals, often leads parents to follow doctors’ recommendations regarding the necessity and timing of proposed treatments. Even when an intersex variation poses no health risks, parents may seek unnecessary medical interventions solely to help their child escape stigmatizing labels.

Although some international medical guidelines and certain recommendations from the Chinese medical community advocate for delaying non-urgent sex assignement surgeries until the child can provide informed consent,^83^ current practices in China still tend to favor early interventions.^84^ Many medical professionals believe that early surgeries help intersex children integrate more smoothly into a binary sex and gender society. In some cases, families are even advised to relocate after ‘gender-normalizing’ procedures are completed.^85^ There is a discernible tendency in Chinese medical practice to prioritize conformity to binary sex and gender norms, which may, at times, come at the expense of fully considering the child’s and parents’ autonomy and informed consent.

## III. LEGAL AND REGULATORY FRAMEWORK IN CHINA

China does not have a single statute specifically addressing intersex status or medical treatment. Instead, relevant norms are found across several sources, including constitutional equality principles, provisions of the Civil Code relating to personality rights and medical decision-making, administrative rules governing health institutions and civil registration, and clinical guidelines issued by medical authorities. These instruments, grounded in a binary understanding of sex and gender, collectively shape the regulatory environment examined in this section of the manuscript. Current Chinese law only permits binary gender registration (ie, male or female) and does not formally recognize non-binary categories.

Nonetheless, certain legal provisions, such as constitutional guarantees of equality and dignity, as well as those safeguarding bodily autonomy and informed consent under China’s Civil Code, apply equally to all citizens and are therefore also applicable to intersex individuals. In addition, some medical and administrative rules, such as the Rules on Gender Reassignment Surgery Clinical Application Management (2022)^86^ and the Ministry of Public Security’s guidelines on gender marker changes in household registration,^87^ were originally designed for transgender individuals and do not fully address the needs of intersex persons. They may, in certain circumstances, provide a potential legal pathway for intersex individuals seeking to update their legal identity and gender markers. Furthermore, labor law provisions addressing gender-based discrimination in employment may offer protections for intersex individuals. This section examines these legal instruments, assessing their applicability and relevance to the rights of intersex persons in China.

### III.A. Constitutional Rights and Fundamental Protections

In the context of the Chinese legal system, the Constitution serves as the fundamental legal framework, safeguarding basic rights for all citizens.^88^ While it does not specifically address the rights of intersex individuals, its provisions on equality and dignity provide a legal foundation that can be used to challenge gender- or sex-based discrimination or exclusion faced by intersex individuals.

For instance, the Constitution guarantees equal rights for all citizens, regardless of ethnicity, race, gender, or other status.^89^ As such, intersex individuals, as Chinese citizens, are entitled to equal protection and treatment. Furthermore, Article 38 of the Constitution explicitly protects personal dignity, prohibiting insult, discrimination, humiliation, or slander of citizens by any means.^90^ In principle, it supports the right of every person, including intersex individuals, to live free from discrimination based on biological traits that do not conform to traditional binary sex categories.

However, to date, there appears to be no publicly reported judgment in China that directly applies constitutional protections to intersex individuals or sex minorities. Even when constitutional principles such as equality or dignity are referenced in judicial decisions, these citations often appear in unrelated cases and function only as interpretive support, with courts relying primarily on ordinary legislation and sectoral laws such as the Civil Code as the actual and operative legal basis for rulings and enforcement.^91^

Whether individuals can successfully invoke constitutional rights in ordinary litigation remains a matter of debate in China. In practice, courts rarely treat constitutional provisions as directly enforceable in judicial decision-making.^92^ As a result, while the Constitution provides an important symbolic and normative framework, its practical use as a legal remedy for intersex individuals remains uncertain and limited.

### III.B. Provisions in the Civil Code

China’s Civil Code, which came into force in 2021, serves as a comprehensive body of law protecting the civil rights of individuals in China.^93^ Although the Civil Code does not specifically mention the rights of intersex individuals, in the absence of legal exception, its provisions, designed to prevent discrimination in civil matters, ensure bodily autonomy and protect against non-consensual or harmful medical interventions, apply to all individuals, which would logically include intersex persons.

However, in the absence of reported judicial decisions applying these provisions specifically to intersex individuals, the scope of their practical application remains uncertain. To assess this more closely, the following section examines several specific provisions in the Civil Code that could be relevant to protect intersex rights.

#### 1. Equality and Non-discrimination

A core principle of China’s Civil Code is the protection of equality, ensuring that all individuals, without exception, enjoy equal legal status and are protected from discrimination in civil affairs. For instance, Article 4 affirms that all civil subjects have equal legal status in civil activities, and Article 14 ensures that all natural persons possess equal civil rights capacity.^94^ Together, these provisions should guarantee that everyone, regardless of sex characteristics, is entitled to the same civil rights and protections.

#### 2. Right to Physical and Mental Health

Article 1004 of China’s Civil Code explicitly protects an individual’s right to physical and mental health.^95^ While this provision is broadly defined, it may serve as a normative basis for challenging medical interventions that could harm intersex individuals physically or psychologically. This is particularly relevant in cases where irreversible procedures are performed solely to conform to binary sex and gender norms, without regard for the individual’s health needs and personal wishes. Even when performed with parental consent, such interventions may arguably conflict with the intersex child’s substantive right to physical and mental health, particularly if the long-term consequences and the child’s evolving will are not adequately considered.

Nevertheless, while intersex adults may assert this right through informed consent and, where necessary, pursue legal remedies for medical harm or malpractice, intersex minors remain largely dependent on parental decisions and lack the legal capacity to independently invoke this right under the current legal framework.^96^ Moreover, legal avenues for redress remain unclear and largely unused if individuals later claim that early irreversible interventions violated their right to physical and mental health.

Although parents are generally presumed to act in the best interests of the child,^97^ Chinese scholars have observed that parental decisions in complex medical contexts are often shaped by social stigma, limited medical knowledge, or deference to medical authority.^98^ For instance, in intersex cases, some parents and medical professionals may justify early surgical interventions as a means of preventing future stigma or bullying. However, international human rights bodies and clinical experts have increasingly warned that such early and irreversible procedures, particularly those lacking clear medical necessity and performed without the consent of the intersex child, may inflict long-term harm, both physically and psychologically.^99^ Past research on intersex-related cases has also shown that parental proxy medical decisions have, in some instances, failed to adequately account for these lasting consequences.^100^ Although there is a dearth of judicial precedents on the matter, we believe these interventions could constitute substantive interference with intersex children’s right to physical and mental health.

Thus, despite the explicit protection offered by Article 1004, its enforceability in intersex-specific contexts remains unclear. It is still uncertain whether the provision can serve, in practice, as a shield against non-essential and non-consensual medical interventions, or as a legal basis for deferring such procedures until intersex children are capable of meaningfully participating in decisions concerning their bodies and identities.

#### 3. Bodily Autonomy and Integrity

Articles 110, 990, and 1003 of China’s Civil Code clearly stipulate that bodily rights cannot be infringed upon by any organization or individual. These provisions affirm an individual’s right to make decisions about their own body, with no one allowed to violate their bodily integrity without regard for their wishes.^101^ They provide a theoretical legal foundation for safeguarding the autonomy of intersex individuals in making medical decisions regarding their bodies, including those related to altering their sex characteristics or undergoing hormone treatments. In principle, these provisions could also support challenges to unnecessary or non-consensual medical interventions that compromise bodily integrity.

These provisions are particularly important for the protection of intersex infants and children, as they may be too young or lack the capacity to make informed decisions about their own bodies. While Chinese law permits parents or guardians to make proxy medical decisions, it remains unclear whether such decisions, especially those affecting bodily integrity, truly reflect the child’s best interests or consider their future or genuine will. In this regard, when postponement poses no risk to health or development, deferring medically unnecessary, irreversible sex-characteristic surgeries until the child is mature enough to meaningfully participate may better reflect the spirit of the Civil Code’s protection of personal autonomy and bodily integrity.^102^

However, in practice, the enforceability of Articles 110, 990, and 1003 remains highly limited. It is unclear how such protections can be activated, especially when parents, acting as legal guardians, agree with physicians on early interventions. There are currently no publicly reported legal cases in China where these provisions have been successfully used to oppose medically unnecessary interventions on intersex minors. Nor is there an established interpretive guidance that would enable courts to apply these provisions specifically to protect intersex children. As such, while the Civil Code offers a potential, albeit limited, legal safeguard, its real-world effectiveness in protecting intersex bodily autonomy remains largely unused and uncertain.

#### 4. Informed Consent

Article 1219 of China’s Civil Code sets forth the procedure for informed consent in medical contexts, requiring healthcare providers to inform patients (or their families) of their medical conditions, proposed treatments, risks, alternatives, and to obtain their informed consent before any medical intervention.^103^

For intersex adults, this requirement can ensure they are properly informed about their health and available medical options, enabling them to assess the risks and benefits of different treatments and make autonomous decisions about whether a medical intervention is truly necessary.

For intersex infants and children who are unable to provide informed consent, Articles 27 and 34 of China’s Civil Code establish that parents or legal guardians, as the legal representatives of their minor children, have the authority to make medical decisions on their behalf.^104^ Meanwhile, Article 35 further clarifies that parents or guardians must prioritize the child’s best interests when making decisions, considering factors such as the child’s age, cognitive development, and true wishes.^105^ In the case of intersex infants or children, this means that parents or guardians must carefully assess the urgency and necessity of the intervention in relation to the child’s health and safety, its potential long-term psychological and physical impacts, and must respect the child’s autonomy in decision-making, in line with the child’s best interests.

While some parents may argue that early surgical interventions help protect the child from future psychological harm or social exclusion, such as bullying due to visible sex characteristics, these justifications remain ethically and clinically contested.^106^ This is particularly due to the lack of long-term clinical evidence supporting the benefits of early surgical interventions, the documented physical and psychological harms of surgeries, and evolving international guidelines recommending deferral of non-urgent procedures.^107^ While applicability remains in doubt, decisions made solely to conform to binary sex and gender norms, particularly when lacking clear medical necessity, may still be at odds with the child’s long-term well-being and, arguably, with the principle of best interests under Article 35.

Notably, while China’s Civil Code establishes a clear standard for informed consent, Chinese health law also recognizes certain exceptions. As previously noted, Chinese clinical practice may allow limited withholding of information under so-called ‘protective medical measures’ in cases where full disclosure is considered harmful to the patient’s well-being (ie, similar to therapeutic privilege in Western ethics). Although this concept has not been explicitly documented or reported in the context of intersex care, it is applied in other areas in clinical practice and may influence how informed consent is implemented in medical cases perceived as sensitive or complex.

Nevertheless, no known cases have been brought under this framework, and its applicability to intersex individuals remains untested. Still, the informed consent provisions in China’s Civil Code may provide a potential legal foundation for safeguarding intersex individuals’ rights and seeking redress in cases of incomplete or misleading disclosure, or non-consensual medical interventions.

#### 5. Medical Malpractice Liability

Chapter 6 of China’s Civil Code clearly stipulates that healthcare providers shall bear legal responsibility for damages caused by negligent or harmful practices.^108^ Article 1219 specifically provides that if healthcare providers fail to follow the informed consent procedure, resulting in harm to the patient, the medical institution shall be liable for compensation.^109^ These liability provisions provide a legal basis for intersex individuals to seek financial compensation in cases where harmful medical practices cause injury or damage, or where medical malpractice occurs due to a failure to obtain proper informed consent.

Although China’s Civil Code does not explicitly address the rights of intersex individuals, it still creates a broad legal framework that can serve as a basis to safeguard intersex rights through provisions related to equality, the right to health, bodily autonomy, informed consent, and medical malpractice liability.

### III.C. Rules on Gender Registration and Gender Marker Change

#### 1. Gender Markers in Identity Registration

In China, resident identity cards are a commonly used form of legal identification. Article 3 of the Law on Resident Identity Cards of China specifies that the items registered on a resident identity card include name, gender, ethnicity, date of birth, household registration address, citizen identification number, photograph, and fingerprint information, etc.^110^ Among these, the citizen identification number serves as a unique, permanent identity code assigned to each citizen.^111^ The 17th digit of this number functions as the gender marker, with odd numbers assigned to males and even numbers to females, without options for non-binary or other gender identities.^112^ For intersex individuals, the gender marker on their resident identity cards must be designated as either male or female, which may not accurately reflect their gender identity or biological sex.

#### 2. Medical and Administrative Procedures for Gender Marker Change

Although China’s gender registration system predominantly follows a binary framework, medical regulations have been established for adults seeking gender affirming interventions (such as transitioning from male to female or female to male), as well as administrative procedures for those wishing to change their gender markers on official identity documents to reflect this transition.

The Rules on Gender Reassignment Surgery Clinical Application Management (2022), issued by China’s National Health Commission, is a key regulatory document governing gender-affirming surgeries in China.^113^ It specifies that medical institutions and practitioners must meet certain professional and technical qualifications. These include having approved medical specialties in plastic surgery or urology, established ethics committees, and physicians with at least ten years of relevant clinical experience. In addition, they must adhere to informed consent procedures when conducting such surgeries. Looking at the patient’s side, individuals seeking gender reassignment must meet several demanding criteria, including having a consistent desire for gender reassignment for at least five years, being over the age of 18, and not being legally married. They must also provide supporting documentation, including a criminal background check from local authorities, a diagnosis of gender dysphoria from a qualified psychiatrist at a Grade-3 Class-A hospital (ie, the highest-level hospitals in China’s healthcare system), a signed request for surgery, and proof that their immediate family has been informed of the planned surgery.

After undergoing gender-affirming surgery, individuals can apply to update their gender marker on official identity documents.^114^ In 2021, China’s Ministry of Public Security issued the Rules for Household Registration and Resident Identity Card Management (Trial), introducing detailed and standardized procedures for updating gender markers following changes in sex characteristics, such as transitioning to male or female.^115^ According to the rules, applicants must provide a gender identification certificate issued by a domestic Grade-3 Class-A hospital or proof from a qualified judicial authentication body. The application is submitted to the local public security bureau for review and approval. As part of the process, the public security bureau must verify biometric data, including facial images and fingerprints, and, if necessary, conduct a home visit or an in-person verification at the applicant’s registered residence community to confirm the authenticity of the request.^116^ Once the gender marker is updated, a new citizen identification number will be issued.

While this framework primarily addresses individuals transitioning between male and female identities through medical procedures, and is designed with transgender individuals in mind, it may also provide a mechanism for intersex individuals to modify their gender markers (only options male or female) on official identity documents if they choose to do so. For example, after voluntarily undergoing medical interventions to align with a male or female biological sex, or when their gender identity differs from what was previously registered before the medical interventions, they can request such changes. However, this framework, which remains difficult to access for many, also does not fully account for the distinct physical characteristics of intersex individuals.

### III.D. Anti-Employment Discrimination

In China, labor laws explicitly prohibit employment discrimination, covering areas such as recruitment, salary, promotion, and workplace treatment. The Labor Law of China, the Employment Promotion Law of China, and the Regulations on Employment Services and Employment Management together form the core legal framework that ensures equal employment opportunities and fair treatment for all workers.^117^ In terms of gender-based employment discrimination, these laws and regulations explicitly prohibit discrimination based on gender, stating that workers must not be treated unfairly on the basis of gender.^118^ While these provisions do not specifically mention intersex individuals, they emphasize the general principles of equality and non-discrimination regardless of one’s gender.

However, given that intersex is not a gender category and is not formally recognized under Chinese law, it remains unclear whether these protections would extend to individuals whose sex characteristics do not align neatly with the binary classification of male or female. Although, as discussed above, there has been at least one judicial case involving gender-nonconforming dismissal, such instances remain rare, and the case in question concerned a transgender individual rather than an intersex person.^119^ There is little evidence that the issue of workplace discrimination against intersex individuals has been addressed in Chinese judicial practice, and no clear guidance has been issued by administrative or judicial bodies on the matter. As such, the applicability of gender-based anti-discrimination provisions to intersex persons may be limited in practice, and their application would likely depend on how an individual is officially registered and perceived in the workplace.

Additionally, China’s labor law framework includes specific provisions to protect female workers from discrimination and unfair treatment. For example, employers are prohibited from refusing to hire women or imposing stricter recruitment standards on them.^120^ Labor contracts cannot include clauses that restrict female workers from marrying or bearing children.^121^ Furthermore, employers are required to prevent workplace sexual harassment of female workers.^122^ While these protections are specifically directed at female workers and do not extend to other gender groups, they may apply, to some extent, to intersex women who are officially registered as female.

For instance, some intersex individuals are assigned and registered as female at birth (despite having intersex traits). In these cases, they will qualify for these protections. By contrast, intersex individuals registered as male or without a gender marker may not have a clear legal basis for invoking such safeguards, even when their gender identification or gender expression aligns more closely with female. Once again, given the lack of reported cases, how these protections might be activated for intersex individuals in practice remains uncertain.

As such, while China’s labor laws prohibit gender-based employment discrimination in general, their capacity to address the specific vulnerabilities of intersex individuals remains limited. Furthermore, employment discrimination safeguards specifically designed for female workers may not be accessible for intersex individuals who are not officially registered as female, regardless of how they self-identify.

## IV. DISCUSSION

While China’s legal framework, in principle, guarantees rights for all citizens, intersex individuals remain largely invisible in national laws, administrative policies, public health initiatives, and broader social discourse in this country.

This legal invisibility is most evident in the absence of statutory terminology. Terms such as ‘intersex,’ ‘DSD,’ or even ‘sex characteristics’ are entirely absent from legal texts. Legally and administratively, intersex persons are subsumed into the binary categories of male or female,^123^ with no formal recognition given to their distinct biological traits. This lack of specific terminology in the law reflects a broader absence of institutional recognition, making it difficult for intersex individuals to be acknowledged as a distinct group with specific preferences and needs. The legal invisibility not only obscures their lived experiences but also impedes their ability to assert personal rights or access protective measures tailored to their circumstances.

Certain legal provisions, such as the constitutional guarantees of equality and non-discrimination for all citizens, and the Civil Code’s protections for bodily autonomy and informed consent, could, in theory, be invoked by intersex individuals. However, the lack of appropriate legal terminology, conceptual clarity, or interpretive guidelines makes it unclear and uncertain when and how, if ever, these broadly formulated protections might apply in practice. Similarly, China’s labor law framework reflects the same limitation. Existing legal texts typically refer to general categories such as ‘all citizens,’ ‘every individual,’ or ‘all employees,’ without addressing the distinct vulnerabilities of people with intersex traits. To date, there are no court decisions or administrative interpretations that explicitly affirm the applicability of these legal protections to intersex individuals.

Even if judicial cases involving intersex individuals were to arise, the absence of clear statutory rules would leave outcomes largely subject to the discretion of individual judges interpreting general legal norms. Given that China’s legal system does not operate on a precedent-based common law model, judges would not be bound to follow previous rulings, particularly when the statutory basis is ambiguous. This compounded uncertainty, arising from both normative gaps and a lack of implementation mechanisms, does not sufficiently provide the safety net that would make intersex individuals feel adequately safe and protected under Chinese law.^124^

This legal ambiguity generates ‘ripple effects’ across multiple levels. Without clear legal standards, ethical oversight, or accountability mechanisms, parents and doctors are often left in the dark on how to address the preferences and needs of this particular group of the population. They are also often left without consistent guidance when making decisions for intersex children. When deciding medical interventions, they rely on traditional social norms of binary sex and paternalism, which do not sufficiently account for long-term welfare considerations of intersex children. Parents of intersex children, operating in an environment with limited legal guidance and substantial social pressure, often make life-altering decisions without access to balanced information or adequate institutional support.

Following on these social norms and the absence of explicit legal protection, violations of intersex rights, including non-consensual surgeries, workplace discrimination, and potential administrative obstacles, are less likely to be reported, documented, or addressed. For instance, in China, there are extremely few publicly available reports, media investigations, or court decisions that address the lived experiences or rights violations of intersex individuals.^125^ This veil of secrecy around Chinese intersex people makes it difficult for them to raise awareness about their variation and advocate for their rights.

This invisibility is compounded by the near-total absence of psychosocial support systems. In particular, there are no formal guidelines requiring that medical decisions involving intersex children consider long-term psychological outcomes or be deferred until the child is capable of providing informed consent. Nor does it offer institutional support, such as publicly funded counseling, peer networks, or psychosocial services that address the emotional and mental health challenges faced by this community. Existing psychological services in China are often limited, underfunded, or focused on acute psychiatric diagnoses.^126^ Intersex individuals and their families lack access to community-based support, dedicated psychosocial services, or safe and supportive environments where their experiences are recognized and affirmed. Medical practice continues to prioritize physical normalization over holistic approaches that foster emotional resilience, identity formation, and social integration.

This systemic legal and institutional neglect contributes to a broader form of social invisibility. In China’s public discourse, intersex issues are also rarely addressed. Educational curricula, mainstream media, and public awareness campaigns seldom mention the existence or rights of intersex individuals. Even in the rare cases where intersex topics are covered, they are often portrayed through a pathologizing or sensationalized lens, reinforcing misunderstanding and stigma. This sustains a cycle of limited public understanding of bodily diversity and contributes to the continued social exclusion of intersex individuals.

In recent years, international human rights bodies have increasingly called for both the recognition and protection of the fundamental rights of intersex individuals. A number of United Nations (UN) bodies, including the UN Committee on the Rights of the Child and the UN Independent Expert on Protection Against Violence and Discrimination Based on Sexual Orientation and Gender Identity, have explicitly recommended that state parties ensure intersex children have the right to their sex and gender identities reflected in legal identification documents.^127^ These recommendations underscore a growing international consensus on the need to enhance the legal visibility of intersex individuals and to affirm their fundamental rights.

In addition to legal recognition, there is a clear international consensus against medically unnecessary and non-consensual procedures on intersex children. These bodies have consistently called on state parties to prohibit such interventions and to ensure that treatment decisions are deferred until the individual can give informed consent, except in cases where deferral would pose a serious risk to health.^128^

Additional safeguards have also been recommended by the UN Committee on the Rights of Persons with Disabilities and the OHCHR, including extending statutes of limitations, strengthening accountability, and ensuring access to fair and effective remedies.^129^ In the area of employment, the UN Human Rights Committee has underscored the need to bridge the gap between law and practice to protect intersex individuals from discrimination, reaffirming that rights recognition must be accompanied by enforceable implementation.^130^

Beyond the UN system, the international momentum is echoed in the positions of professional and multilateral organizations such as the World Health Organization (WHO), the World Medical Association (WMA), and the International Labour Organization (ILO). These bodies emphasize the importance of respecting intersex individuals’ bodily autonomy, prohibiting non-consensual medical interventions, upholding ethical standards in clinical care, and addressing workplace discrimination, exclusion, and harassment.^131^

Together, these developments reflect a growing global consensus on the need for enforceable legal protections, ethical medical practices, and inclusive support systems.

Meanwhile, an increasing number of jurisdictions have begun to acknowledge the existence of intersex individuals and have adopted legal measures to enhance their recognition, autonomy, and protection. The comparative examples included here are not intended as a comprehensive survey but as illustrative models highlighting different regulatory approaches to intersex medical treatment and legal recognition. They are used to identify concrete reform options that may be adaptable, with appropriate contextualization, to the Chinese legal system.

For instance, Malta is widely regarded as a global leader in protecting gender diversity through a human-rights-based framework. The Gender Identity, Gender Expression and Sex Characteristics Act (2015) allows individuals to amend gender markers in civil records based on self-determination and explicitly protects persons with variations of sex characteristics from discrimination and from non-consensual medical interventions.^132^ The law further strengthens these protections through enforceable sanctions, including criminal penalties for violations. For China, the key lesson is the value of establishing clear legal safeguards that prioritize bodily integrity and informed consent in the medical management of intersex variations. Similarly, Portugal and Argentina have adopted rights-based approaches to gender diversity grounded in the principle of self-determination. Portugal’s Law No. 38/2018 and Argentina’s Gender Identity Law both recognize the right to legal gender recognition based on self-identified gender identity.^133^ Notably, Portugal’s legislation also provides explicit protections for individuals’ primary and secondary sex characteristics, reinforcing safeguards against medically unnecessary interventions. For China, these examples highlight the importance of linking legal gender recognition frameworks with protections for bodily integrity and autonomy.

In Australia, protections for intersex individuals have also progressively expanded. Amendments to the Sex Discrimination Act (1984) in 2013 extended anti-discrimination coverage to intersex status.^134^ In 2022, the Fair Work Act was revised to prohibit adverse employment actions on this basis,^135^ offering a clear legal pathway to challenge workplace discrimination. Additionally, legislation introduced in the Australian Capital Territory in 2023 specifically focused on safeguarding intersex rights in medical settings.^136^ Similarly, Greece, Spain, and Germany have passed laws strictly limiting or banning non-consensual medical procedures on intersex minors, affirming their legal rights to bodily integrity, informed consent, and personal autonomy in healthcare decisions.^137^ Germany, after extended debate, has also passed legislation explicitly recognizing intersex individuals’ rights to self-determination and bodily autonomy.^138^ This development was significantly shaped by case law, including the 2008 Völling case and the 2015 Michaela Raab case, both of which involved non-consensual and irreversible medical interventions that resulted in serious harm.^139^ For China, these reforms illustrate how legal protection for intersex individuals may be progressively strengthened through targeted legislative amendments in specific domains, especially employment and medical care.

In addition to these international examples, developments within the Hong Kong Special Administrative Region, a distinct jurisdiction operating under China’s ‘one country, two systems’ framework, offer regionally relevant practices that may inform reform efforts in mainland China. In November 2024, the Hong Kong Legislative Council issued a written reply to a question from a legislative councilor regarding the protection of intersex persons in Hong Kong, titled ‘Protecting Rights and Interests of Intersex Persons.’^140^

The reply included official statistics on the number of infants diagnosed with indeterminate sex over the past decade, although these figures were described as for general reference only. It also outlined the current protection measures in place for intersex persons in Hong Kong. These measures include a multidisciplinary clinical approach in which hospital teams evaluate each case based on the anticipated sex development and functional prognosis of the child, and reach treatment decisions in full consultation with the parents. Considerations include diagnostic findings, the degree of masculinization due to prenatal androgen exposure, hormonal responsiveness, potential sexual and reproductive function, and the risk of severe or life-threatening complications.

As outlined in the reply, all relevant medical decisions are made on an individualized basis in Hong Kong, with the best interests of the child as the guiding principle. Furthermore, the reply noted that, in clinical practice, physicians are encouraged to promote open communication between parents and children about intersex variations and to ensure that families are fully informed about available medical options. In addition, Hong Kong’s Department of Health and the Hospital Authority have developed sensitivity training materials for healthcare professionals, emphasizing the importance of obtaining informed consent prior to conducting physical examinations on intersex individuals. While these initiatives remain administrative rather than legislative in nature and do not yet constitute a formal set of best practice guidelines, they nonetheless reflect a growing recognition of intersex rights in the region.

In contrast, mainland China has yet to formally engage in similar legal reforms or introduce specific measures aimed at protecting intersex individuals. To date, there is little indication that the government has considered reviewing existing laws or policies to address the specific needs of this population. This ongoing absence highlights a potential gap between domestic legal frameworks and evolving international human rights standards, which may leave this vulnerable population without formal recognition or adequate protection in this country.

As a state party to several international human rights treaties, including the Convention on the Rights of the Child,^141^ China has incorporated the principle of the ‘best interests of the child’ into its domestic legal system, such as the Law on the Protection of Minors of China and the Law on the Promotion of Family Education of China.^142^ However, recent recommendations issued by the UN Committee on the Rights of the Child concerning the protection of intersex children’s human rights have yet to be reflected in China’s national legislation or public policy. This may be partly due to limited societal awareness and a prevailing unfamiliarity with the notions of this population and sexual diversity beyond binary sex and gender. Discussions around sex and gender diversity, including intersex people, remain a rare occurrence.

In such contexts, a phased or incremental approach may be the most feasible option to increase the visibility of intersex people and facilitate the understanding of their particular challenges. Foundational steps such as government-led data collection, public consultation, education, and awareness-raising campaigns are essential to create an enabling environment for future reform. Chinese authorities could begin by conducting policy reviews and feasibility studies to assess the legal, medical, and social needs of intersex populations.

At the same time, public awareness campaigns through popular media and e-platforms would serve as an important early step. These efforts could be supported by using inclusive and neutral terminology in public health materials and media. In particular, efforts should focus on promoting a non-pathologizing understanding of intersex traits. This could involve replacing medicalized and stigmatizing terms, such as ‘DSD’ with language that affirms bodily diversity and respects individual identity. Shifting the public narrative in this way is a crucial step toward reducing stigma and fostering broader societal acceptance.

Inclusive educational curricula and professional training for healthcare providers (especially in obstetrics, pediatrics, and genetics), including the development of standards of good practices, will play a critical role in challenging misconceptions, correcting misinformation, and increasing the visibility of intersex persons in society.

China could prioritize the development of professional best practices for intersex care. These standards, developed and implemented by healthcare professionals, can help guide clinical decision-making in advance of formal legal codification and offer immediate protections within clinical settings.

Key best practice elements for medical professionals should include: (i) Deferring non-urgent and irreversible procedures until intersex minors are capable of providing informed consent; (ii) Establishing a minimum age of consent for such procedures, drawing on international best practices (many of which recommend deferral until at least the age of 12–16, depending on individual degree of maturity)^143^ and relevant provisions in China’s Civil Code on minors’ civil capacity;^144^ (iii) Clarifying limitations on doctors’ therapeutic privilege, including the obligation to systematically disclose medical information about an intersex variation to parents; (iv) Replacing stigmatizing or pathologizing terminology, such as ‘DSD’, with neutral, affirming language in medical records, health education, and public communications; and (v) Considering introducing mandatory clinical ethics consultation and review mechanisms for major medical decisions involving intersex minors.

Recent scholarship indicates that ethics consultations are emerging as an important mechanism for addressing ethical challenges in clinical decision-making in China.^145^ However, a widely institutionalized system of full-time ethicists has not yet been widely established in this country.^146^ At present, in Chinese hospitals, ethics committees are typically internal bodies responsible for addressing ethical issues, with their primary functions focused on the review of biomedical research and the clinical application of new medical technologies.^147^ Chinese scholars have nevertheless recommended expanding their role to include consultation on significant treatment decisions for minors, particularly those with long-term consequences for a child’s physical and psychological well-being.^148^ In this context, for intersex care, a two-tiered approach could therefore be considered. As a first step, the attending physician could be required to consult with a qualified clinical ethicist (where available) or with an ethics committee member possessing relevant training, and to ensure that such consultations are properly documented for record-keeping and accountability purposes. For more complex cases, particularly those involving irreversible surgical interventions with potentially long-term consequences, the consultation could be elevated to the full ethics committee for interdisciplinary review. Such review bodies should include, in addition to medical professionals and ethicists, experts such as child psychologists. This structure would help align decisions with the intersex child’s evolving capacities and best interests.

As professional norms and public awareness evolve and stabilize, these practices can both inform the development of regulatory frameworks and be incorporated into future legislation.

In the medium to long term, China could draw on international models discussed above to guide its legal reforms. Jurisdictions such as Malta and Australia have recognized intersex status and acknowledge ‘sex characteristics’ as a protected category. These reforms, on one hand, strengthen legal protections, and on the other hand, significantly promote the social visibility and recognition of intersex individuals. Visibility, in this sense, is not merely symbolic. It affirms intersex persons as legitimate rights-holders and subjects of legal concern, helping to counter longstanding cultural erasure and marginalization.

As public understanding increases, Chinese lawmakers could consider revising existing laws and administrative frameworks and, where appropriate, encouraging the Supreme People’s Court of China to issue authoritative judicial interpretations^149^ to clarify the applicability of existing legal protections to intersex individuals. Without explicit recognition in statutory language, existing legal rights risk remaining symbolic and unenforceable.

Eventually, legal safeguards may be introduced to prohibit medically unnecessary procedures on intersex minors. These could include a legally defined minimum age of consent for non-urgent interventions, explicit protections for bodily autonomy and informed consent, and mechanisms for oversight and accountability. Where appropriate, these protections should be accompanied by enforceable remedies for rights violations, including administrative sanctions, fines, or even possible jail sentences in case of non-consensual surgery.

However, legal reform alone is insufficient. International best practices have also increasingly emphasized the need for comprehensive psychosocial support. For instance, the UN Committee on the Rights of Persons with Disabilities has recommended that state parties provide adequate counseling and support for families of intersex children and offer long-term psychosocial care for intersex individuals who have experienced non-consensual medical interventions.^150^ Similarly, in 2021, the Australian Human Rights Commission called for not only legal protections for bodily autonomy, but also significant investment in peer and family support networks.^151^ Following this, in 2023, the Government of the Australian Capital Territory enacted legislation establishing oversight mechanisms for non-urgent medical procedures on individuals with variations in sex characteristics. Crucially, it was accompanied by substantial funding for psychosocial services for affected individuals and their families.^152^

For jurisdictions without explicit intersex legislation, Canada offers a useful reference point in how psychosocial support and inclusive health communication can begin to address the needs of intersex individuals. Although Canada does not currently have national legislation specifically protecting intersex persons, it has made incremental progress in fostering more affirming healthcare environments. Recent scholarship highlights the growing recognition of the need for compassionate, non-pathologizing communication in clinical settings, which has been described as ‘a quality of heart, of presence, and of really caring,’ that emphasizes empathy, validation, and trust-building over correction or normalization.^153^ Civil society organizations in Canada have also played a pivotal role in advancing psychosocial support for intersex individuals. Organizations such as Egale Canada and Intersex Canada have been actively involved in advocacy, public education, and peer support initiatives.^154^ These groups work to raise awareness about the lived experiences of intersex people, combat stigma, and provide safe, affirming spaces for intersex individuals and their families. Some community-led projects offer online resources, support groups, and workshops aimed at empowering intersex youth and educating professionals in healthcare and education.

As a result, intersex individuals in Canada have greater access to peer support and affirming communities—something still largely lacking in the Chinese context. This demonstrates a viable pathway toward progress that, even in the absence of explicit legal protections and social support systems, can begin to alleviate the social pressures and structural inequalities faced by intersex individuals.

Psychosocial support for intersex individuals and their families remains minimal in China. Given that legal reform and formal interpretive guidance may take time to materialize, and that broader societal recognition also requires gradual progress, advancing psychosocial support systems may offer a more immediate and tangible way to improve the lived experiences of intersex individuals. A coordinated and inclusive approach to intersex care can prioritize the development of accessible community-based mental health services, the integration of intersex-related training for counselors and healthcare professionals, and the creation of peer support networks that affirm the inherent value of diverse gender identities and bodily experiences in society. Public awareness campaigns and inclusive educational programs in schools can also help reduce bullying and support identity development from an early age. These efforts would not only provide immediate relief to intersex individuals and their families but also help lay a stronger social foundation for future legal and institutional reform.

Within the Chinese context, Hong Kong offers a relevant regional example of community-led progress. Organizations such as PrideLab and the Family Planning Association of Hong Kong, along with the work of activist Small Luk (an intersex rights advocate), have been working to raise awareness and build support for intersex individuals, even in the absence of sweeping legislative change.^155^ These efforts, while still evolving, have brought increased visibility to intersex issues in local media and public forums.

In addition to learning from Western jurisdictions and local practices in Hong Kong, China could also benefit from engaging with regional movements that reflect shared cultural and social contexts. For instance, the 2023 Asian Intersex Statement, issued by Intersex Asia, outlines an Asia-specific vision for affirming bodily autonomy, eliminating non-consensual interventions, and promoting community-based psychosocial support.^156^ While China is not yet a member of this regional organization, increased awareness of and engagement with such initiatives may help foster a respectful, culturally informed model of intersex care. Integrating lessons from Asian advocacy movements could offer a promising pathway toward building inclusive and sustainable support structures in the Chinese context, even before comprehensive legal reform is in place. Moreover, greater publicity and outreach could also enable Chinese intersex individuals to connect with regional peer communities, including through joining networks like Intersex Asia, thereby gaining more psychosocial support and benefiting from shared advocacy efforts.

Ultimately, achieving holistic intersex care requires moving beyond a purely medicalized model toward one that affirms dignity, autonomy, and bodily diversity, and provides lifelong psychosocial support.

## V. CONCLUSION

As international consensus continues to grow around the need for explicit recognition and protection for intersex individuals, China remains at a critical crossroads. Intersex individuals in China continue to face profound challenges stemming from cultural invisibility, limited legal recognition, and medical practices that prioritize normalization over autonomy and gender diversity. Despite formal guarantees of equality, bodily integrity, and informed consent in China’s Constitution and Civil Code, it remains uncertain whether these rights can be meaningfully applied to intersex individuals. Public awareness of intersex issues remains very limited. Medical decision-making, particularly in childhood medical interventions, is frequently influenced by binary sex and gender norms, parental authority, and pathologizing frameworks, rather than prioritizing the child’s best interests, autonomy, and evolving capacity to participate in decisions about their own body.

Shifting toward a more inclusive and non-pathologizing view of intersex traits will require time, education, and institutional support. Nevertheless, drawing on emerging international practices, China can begin building a more inclusive and rights-based framework through concrete and incremental steps. Foremost among these may be the development of professional norms and clinical guidelines that clearly recommend deferring medically unnecessary interventions on intersex minors. Establishing such good practices should be a top priority, with responsibility placed on medical professionals, ethics committees, policy researchers, and affected communities to lead this process. Additional measures may include launching public awareness-raising campaigns, incorporating terms such as ‘intersex’ or ‘sex characteristics’ into legal and administrative language, and clarifying the applicability of existing civil rights and anti-discrimination protections. In parallel, it may be possible to envision future reforms to the identity registration system that allow for greater flexibility in sex and gender markers based on a more inclusive, science-based perspective of sex and gender.

In the longer term, ensuring the rights of intersex individuals will depend not only on legal reform, but also on broader cultural and institutional change. Efforts to promote visibility, affirm diversity, and provide psychosocial support should accompany the legislative progress. Guaranteeing that all individuals, regardless of sex characteristics, can live with dignity, autonomy, and equal protection under the law is not only a matter of justice and ethics, but also a meaningful step toward a more inclusive and respectful society.

## Footnotes

1 For example, the development of genetic testing technology has enabled individuals to gain deeper insights into their sexual characteristics and physiological status. See Tinca J.C. Polderman et al., *The Biological Contributions to Gender Identity and Gender Diversity: Bringing Data to the Table*, 48 Behav. Genet. 95–108 (2018); Katie M. Saulnier et al., *Prenatal Genetic Testing for Intersex Conditions in Canada*, 43 J. Obstet. and  Gynaecol. Can. 369–371 (2021); Furthermore, advancements in technology, particularly the proliferation of the internet and social media, have facilitated rapid access to information about gender identity and gender expression. See Chadwick K. Campbell et al., “*I was always trying to figure it out… on my own terms”: Structural Barriers, the Internet, and Sexual Identity Development among Lesbian, Gay, Bisexual, and Queer People of Different Generations*, 70 J. Homosex. 2560–2582 (2023); Additionally, human rights organizations play a pivotal role in advocating for gender expression and gender identity rights. See Meredith L. Weiss, *Building Solidarity on the Margins: Seeking SOGIE Rights in ASEAN*, 20 J. Hum. Rights 194–210 (2021); Cynthia Burack, *Sexual Orientation and Gender Identity (SOGI) Human Rights Assistance in the Time of Trump*, 14 Politics  Gend. 561–580 (2018); Georgiann Davis & Maddie J. Evans, *Surgically Shaping Sex: A Gender Structure Analysis of the Violation of Intersex People’s Human Rights*, in 2 Handbook of the  Sociology of  Gender 273–284, (Barbara J. Risman et al., eds., 2018).

2 Tiffany Jones & Anita Jacombs, *Intersex and Sexuality Education: Editorial Introduction*, 21 Sex  Educ. 497–503 (2021); Markus Bauer et al., *Intersex Human Rights*, Int. J. Hum. Rights 724–749 (2019).

3 The Office of the United Nations High Commissioner for Human Rights, *Intersex People*, https://www.ohchr.org/en/sexual-orientation-and-gender-identity/intersex-people (accessed Apr. 30, 2025).

4
Tiffany  Jones et al., Intersex: Stories and  Statistics from  Australia (Open Book Publishers) (2016).

5 For instance, variations like PAIS are often identified at birth or early childhood due to atypical genitalia, while CAIS is typically diagnosed later, often in adolescence, when individuals may present with the absence of menstruation. CAH can cause severe medical complications, such as abnormal heart rate and hypoglycemia, necessitating urgent treatment. In contrast, Klinefelter Syndrome, characterized by reduced facial and body hair or underdeveloped genitalia, is often not realized by their families or even by the individuals within the intersex community themselves. See *Id*. and Fae  Garland & Mitchell  Travis, Intersex  Embodiment: Legal  Frameworks beyond  Identity and  Disorder (Bristol University Press) (2022).

6 Peter Hegarty et al., *Understanding of Intersex: The Meanings of Umbrella Terms and Opinions about Medical and Social Responses among Laypeople in the United States and United Kingdom*, 8 Psychol. Sex. Orientat. Gend. Divers. 25–37(2021); InterACT, *InterACT Statement on Intersex Terminology*, https://interactadvocates.org/interact-statement-on-intersex-terminology/ (accessed Apr. 30, 2025).

7 Small Luk, *Beyond Boundaries*, Intersex  Day, https://intersexday.org/en/beyond-boundaries-intersex-hk-china/ (accessed Mar. 13, 2026); Liu Minghui, *Legal and Policy Research on Ensuring Equal Employment Rights for Sexual Minority Groups Under the UNDP “Being LGBTI in Asia” Project*, UNDP, https://cnlgbtdata.com/files/uploads/2021/05/%E4%BF%9D%E9%9A%9C%E6%80%A7%E5%B0%91%E6%95%B0%E7%BE%A4%E4%BD%93%E5%B9%B3%E7%AD%89%E5%B0%B1%E4%B8%9A%E6%9D%83%E7%9A%84.pdf (accessed Mar. 13, 2026).

8 See Peter Hegarty et al., *supra* note 6; InterACT, *supra* note 6.

9 Trove Lundberg et al., *Making Sense of “Intersex” and “DSD”: How Laypeople Understand and Use Terminology*, 9 Psychol. Sex. 161–173 (2018); Terese Knoppers et al., *A Quality of Heart, of Presence, and of Really Caring: Toward Affirmative Intersex Health Communication in Canada*, 12 Front. Public  Health (2024).

10 InterACT, *Australia Passes Landmark Anti-Discrimination Legislation Officially Protecting Intersex People*, https://interactadvocates.org/australia-passes-landmark-anti-discrimination-legislation-officially-protecting-intersex-people/ (accessed Apr. 30, 2025); Human Rights Watch, *I Want to be like Nature Made Me: Medically Unnecessary Surgeries on Intersex Children in the US*, https://www.hrw.org/report/2017/07/25/i-want-be-nature-made-me/medically-unnecessary-surgeries-intersex-children-us (accessed Apr. 30, 2025).

11 For instance, many studies have examined the status and efforts regarding intersex rights in regions such as Europe, Canada, the United States, and New Zealand, etc. Australia and Malta also have legislation in this regard. See James Garland & Santa Slokenberga, *Protecting the Rights of Children with Intersex Conditions from Nonconsensual Gender-Conforming Medical Interventions: The View from Europe*, 27 Med. Law  Rev. 482–508 (2019); Katie Saulnier et al., *Harnessing Law and Policy for the Protection of Intersex Individuals: Facing Up to Lacunae in Canadian Legal Frameworks Against Sex and Gender*, 15 McGill J. Law  Health 207–258 (2023); Juan C. Jorge et al., *Intersex Care in the United States and International Standards of Human Rights*, 16 Glob. Public  Health 679–691 (2021); Claire Breen & Katerina Roen, *The Rights of Intersex Children in Aotearoa New Zealand*, 31 Int. J. Child. Rights 533–567 (2023); Sex Discrimination Amendment (Sexual Orientation, Gender Identity and Intersex Status) Act 2013, Australia’s Federal Register of Legislation, https://www.legislation.gov.au/C2013A00098/latest/text (accessed Apr. 30, 2025); Gender Identity, Gender Expression and Sex Characteristics Act 2015, Leġiżlazzjoni Malta, https://legislation.mt/eli/cap/540/eng/pdf (accessed Apr. 30, 2025).

12 UNDP, *Report on the Living Conditions of Sexual Minorities in China—A Survey on Social Attitudes Based on Sexual Orientation, Gender Identity, and Gender Expression*, https://www.undp.org/sites/g/files/zskgke326/files/migration/asia_pacific_rbap/UNDP-CH-PEG-Being-LGBT-in-China_CH.pdf (accessed Apr. 30, 2025); Qian Siwen, *Special Report: The Situation of Sexual and Gender Minorities in China Needs Improvement—Interview with Professor Liu Minghui from China Women’s University*, UN News, June 24, 2019, https://news.un.org/zh/story/2019/06/1036281 (accessed Apr. 30, 2025); Amnesty International, *China: I Need my Parents’ Consent to be Myself: Barriers to Gender-Affirming Treatments for Transgender People in China*, https://www.amnesty.org/en/documents/asa17/0269/2019/en/ (accessed Apr. 30, 2025); CommonLanguage, *The Rights Situation of Transgender and Intersex People in Mainland China: A Study of Media Coverage*, https://www.tycommonlanguage.com/post/%E4%B8%AD%E5%9B%BD%E5%A4%A7%E9%99%86%E8%B7%A8%E6%80%A7%E5%88%AB%E4%B8%8E%E9%97%B4%E6%80%A7%E4%BA%BA%E7%9A%84%E6%9D%83%E5%88%A9%E7%8A%B6%E5%86%B5-%E5%AF%B9%E5%AA%92%E4%BD%93%E6%8A%A5%E9%81%93%E7%9A%84%E7%A0%94%E7%A9%B6 (accessed Apr. 30, 2025).

13 Under the “One Country, Two Systems” framework, Hong Kong operates as a Special Administrative Region (SAR) of China with a high degree of autonomy. While areas such as diplomacy and national defense remain under the authority of China’s central government, Hong Kong maintains independent control over its legal system, public health policies, and civil society governance. This arrangement allows the region to develop policies and advocacy initiatives that may differ from those in mainland China. See Basic Law, *Hong Kong’s Constitutional and Mainland Affairs Bureau*, https://www.basiclaw.gov.hk/en/basiclaw/index.html (accessed Apr. 30, 2025); The State Council of the People’s Republic of China, *The Practice of the “One Country, Two Systems” Policy in the Hong Kong Special Administrative Region*, https://english.www.gov.cn/archive/white_paper/2014/08/23/content_281474982986578.htm (accessed Apr. 30, 2025).

14 Government of the Hong Kong Special Administrative Region*, LCQ19: Protecting Rights and Interests of Intersex Persons*, https://www.info.gov.hk/gia/general/202411/20/P2024112000299.htm (accessed Apr. 30, 2025); PrideLab, *Understanding the Intersex Flag*, https://www.pridelab.hk/2022/projects/infodesign/3510/ (accessed Apr. 30, 2025); Family Planning Association of Hong Kong, *Understanding Intersex Persons*, https://www.famplan.org.hk/zh/resources/feature-articles/detail/ej20141231 (accessed Apr. 30, 2025).

15 Lu Lin, *Analysis of Informed Consent in Intersex Surgical Intervention and Postmodern Philosophical Thinking*, 44 Med. Philos. 15–19 (2023); Hu Sha, *Construction of Intersex Gender in Criminal Law*, 23 Chin. J. Hum. Sex. 103–107 (2014); Li Y., *Critique and Reconstruction of the Gender Binary Paradigm in Law: A Narrative from the Perspective of Intersex Individuals*, 19 Law  Soc. Dev. 10–20 (2013).

16 See Lu, *supra* note 15; CommonLanguage, *supra* note 12; Winston Luhur W et al., *Public Attitudes Toward LGBTI Rights: China*, UCLA Williams  Institute, https://williamsinstitute.law.ucla.edu/wp-content/uploads/Public-Opinion-Trans-China-Chinese-Jun-2021.pdf (accessed Apr. 30, 2025).

17 The intersection between intersex individuals and judicial practice in China is documented exclusively in academic literature, and even these discussions are limited to cases involving sexual violence and human trafficking. In publicly available judicial rulings and officially recorded legal cases, references to intersex characteristics or identities are virtually nonexistent. See Hu, *supra* note 15; Li, *supra* note 15.

18
*Id*.

19 As past research indicates, the substantial variability between these estimates (0.05%–1.7%) arises primarily from differing definitions and scope used to classify intersex variations. Broader estimates (1.7%) typically encompass a wide range of conditions, including chromosomal variations (eg Klinefelter or Turner syndromes), hormonal differences, and gonadal anomalies, some of which may not be externally evident at birth. Narrower estimates (around 0.05%) usually focus solely on variations with visible genital differences identifiable at birth. See Garland & Travis, *supra* note 5.

20 The Office of the United Nations High Commissioner for Human Rights, *A Step Forward for Intersex Visibility and Human Rights*, https://www.ohchr.org/en/stories/2015/09/step-forward-intersex-visibility-and-human-rights (accessed Apr. 30, 2025); InterACT, *What is Intersex?,*  https://bloomforall.com/parent/what-is-intersex/ (accessed Apr. 30, 2025).

21 Chan Sin-Yee, *Would Confucianism Allow Two Men to Share a Peach? Compatibility between Ancient Confucianism and Homosexuality*, in The  Bloomsbury  Research  Handbook of  Chinese  Philosophy and  Gender, 173–202 (Ann A. Pang-White, ed., 2016); Zhu Ziyu & Li Mengxin, *Gender Differences and Their Implications: How Folklore Shapes Gender Roles*, Dazhong  Daily  Press, Oct. 10, 2021, https://dzrb.dzng.com/articleContent/1158_920059.html (accessed Apr. 30, 2025); Ji Yingchun & Zheng Zhenzhen, *China’s Low Fertility Rate from the Perspective of Gender and Development*, 8 Soc. Sci. Chin. 143–161, 207–208 (2018); Yenching Academy of Peking University, *Coexistence of Traditional Cultural Influence and the Pursuit of Individual Freedom: Professor Xie Yu on Family Changes in Contemporary China*, https://yenching.pku.edu.cn/info/1039/3691.htm (accessed Apr. 30, 2025).

22
*Id*; Wang Yuanyuan et al., *Mapping Out a Spectrum of the Chinese Public’s Discrimination Toward the LGBT Community: Results from a National Survey*, 20 BMC Public  Health. 669 (2020).

23 Confucian culture itself places strong value on conformity and deference to established norms. These values have contributed to a social ethos that prioritizes tradition, role stability, and adherence to normative expectations. Within this framework, individuals are generally expected to conform to one of the two traditionally recognized sexes and genders (ie, male or female), leaving limited space for those who do not fit neatly within this binary structure. See Wang et al., *supra* note 22; Zhu & Li, *supra* note 21; Helal U. Ahmed & Zhang Jielin, *A Behavioral Study on the Influences of Confucianism in Chinese Society*, 61 Philos. Prog. 1–2 (2017).

24 Amnesty International, *supra* note 12; Lu, *supra* note 15; Hu, *supra* note 15; Li, *supra* note 15.

25 See Lu, *supra* note 15; A typical illustration of how sociocultural perceptions impact intersex care in China is found in a recent clinical case reported by a hospital in Shanghai in April 2025. The case involves a 13-year-old intersex patient who underwent gonadectomy and genital reconstruction, not due to urgent medical necessity, but rather based on the strong wishes of the patient and her family to align her body with a female identity shaped by her upbringing. According to the report, the patient’s physical health was not affected by her intersex traits. Instead, the decision to pursue surgery was largely motivated by a desire to avoid the stigma associated with intersex traits and future social exclusion. The attending physician reportedly also supported early intervention to minimize potential psychological distress from public judgment or shame. Although the procedure followed clinical protocols and involved the patient’s consent, it reflects the enduring influence of binary sex and gender norms on the care of intersex minors in China. See Ye H., *Remodeling the New Body, Recreating the New Life*, https://www.shkjb.com/content.html?id=238174 (accessed Apr. 30, 2025).

26 See Lu, *supra* note 15; Hu, *supra* note 15; Li, *supra* note 15; Group of Urological Surgery, Branch of Pediatric Surgery, Chinese Medical Association, *Chinese Expert Consensus on Disorders of Sexual Development*, 40 Chin. J. Pediatr. Surg. 289–297 (2019).

27 See Lu, *supra* note 15; Xiao Tie, *Xixi Teacher: Let More People See the Completeness of Intersex Individuals*, https://zhuanlan.zhihu.com/p/24496877 (accessed Apr. 30, 2025).

28 UNDP & China Women’s University, *Legal Recognition of Gender Identity for Transgender Individuals: Assessment of Relevant Laws and Policies in China*, https://www.undp.org/sites/g/files/zskgke326/files/migration/asia_pacific_rbap/81e35c0bbe5b80d746519d11bb00d20cb33c8990b529c6664137bf2127ce73cd.pdf (accessed Apr. 30, 2025); Zhang Yong & Gao Yi, *Interview: Mr C, the First Case of Employment Discrimination Against a Transgender Individual in China*, BBC News, Apr. 12, 2016, https://www.bbc.com/zhongwen/simp/china/2016/04/160412_china_transgender_case (accessed Apr. 30, 2025).

29 As previously noted and supported by existing studies, Confucianism, as a philosophical system emphasizing social harmony and order, promotes a rigid alignment between sex, gender, and social roles. In this model, male and female identities are exclusively assigned distinct roles and responsibilities, leaving limited space for individuals who do not conform to this binary. See Chan, *supra* note 21; Ahmed & Zhang, *supra* note 23; Zhu & Li, *supra* note 21.

30 Li, *supra* note 15.

31 As previous studies have confirmed, ancient Chinese medical knowledge and information were often monopolized by specific social classes and primarily transmitted through oral tradition or private teaching, which significantly limited their circulation. Additionally, most medical texts were highly specialized, rendering them inaccessible or difficult for the general public to comprehend. See Lv W., *Medical Knowledge Transmission and Doctors in Sui and Tang Dynasties*, 1 Int. Med. Historical  Rev. 19–39 (2020); Hou Y., *The Research on the Folk Inheritance of Traditional Chinese Medicine: Based on the Source of Literary Sketches, Medical Books and Official History*, 7 Med. Philos. 72–76 (2022).

32 Hu, *supra* note 15; Li, *supra* note 15.

33 UNDP, *supra* note 12; CommonLanguage, *supra* note 12; Zhou Peiqin, *Cisgender: Match between Sex and Gender and its Implications*, 3 Jianghai  Acad. J. 97–104, 254–255 (2020); Lin Hong, *Thinking Gender from an Anthropological Perspective: Taking Intersex as an Example*, 3 J. Xiamen  Univ. (Arts & Social  Sciences) 57–62 (2012).

34 CommonLanguage, *supra* note 12; Lan Tianming, *The Third Type of Toilet Appears in Beijing*, China  Youth  Daily, Aug. 3, 2016, https://zqb.cyol.com/html/2016-08/03/nw.D110000zgqnb_20160803_2-09.htm (accessed Apr. 30, 2025); Zhang Feng*, Expelled from the Bathroom*, The  Paper, Nov. 28, 2020, https://m.thepaper.cn/newsDetail_forward_10181708 (accessed Apr. 30, 2025); Sun Qingyun, *Individual Publicly Claims Intersex Identity: “I Want to Face this Bravely” and Thanks Online Support*, The  Paper, Feb. 9, 2021, https://www.thepaper.cn/newsDetail_forward_11277607 (accessed Apr. 30, 2025).

35 Lin Qiqing, *Why has Beijing Seen a Surge of Gender-Friendly Toilets?*, Curiosity  Daily, Jun. 14, 2016, https://www.guancha.cn/society/2016_06_14_364050.shtml (accessed Apr. 30, 2025).

36 See UNDP, *supra* note 12; Zhang, *supra* note 34, Lin, *supra* note 35; Nanqiandai, *Science Literacy: Common Questions about Intersex People*, https://www.bilibili.com/opus/540915345266920777 (accessed Apr. 30, 2025).

37 Zhang, *supra* note 34; Nanqiandai, *supra* note 36.

38 See Ye, *supra* note 25; In addition, due to the current lack of local research specifically examining healthcare accessibility for intersex individuals in China, existing discussions on transgender populations are used here as supporting evidence. These references highlight the challenges faced by sexual and gender minorities in accessing appropriate healthcare services in China. See Tian Dongliang, *A Gynecologist Opens a Transgender Medical Clinic*, Open  Sina  News, Sep. 8, 2024, https://finance.sina.cn/2024-09-08/detail-incnmzsa6529072.d.html?oid=%E4%B9%90%E5%8A%A8%E4%B8%8B%E6%B3%A8%20 (accessed Apr. 30, 2025); Chen Xiaoyan, *In Gender-Nonconforming Adolescent Clinics, the Abused Doctors and Torn Families*, True  Story  Project, Jan. 8, 2023, https://mp.weixin.qq.com/s?__biz=Mzk2NDgxMzE3MQ==&mid=2247690136&idx=1&sn=358da9a554cc4616badeaef512bbc687&source=41#wechat_redirect (accessed Apr. 30, 2025).

39 CommonLanguage, *supra* note 12.

40 CommonLanguage, *supra* note 12; Liu Wanyuan, *Pan Bolin: Gender-Nonconforming Treatment is Rare, but They Need Medical Support*, The  Paper, Aug. 19, 2020, https://www.thepaper.cn/newsDetail_forward_8786958 (accessed Apr. 30, 2025).

41 UNDP, *supra* note 12; CommonLanguage, *supra* note 12.

42 Hu, *supra* note 15; Li, *supra* note 15; Lin, *supra* note 35.

43 Chongqing Morning Post, *Woman Becomes Intersex: Possesses Two Reproductive Systems, a Rare Case in the Country*, https://news.sina.cn/sa/2005-01-11/detail-ikknscsi5479858.d.html (accessed Apr. 30, 2025); Chi Ming, *Chongqing Man Diagnosed with Pseudo-Hermaphroditism Lives a “Dual-Gender” Life for 22 years*, Beijing  Times, Nov. 9, 2015, http://www.xinhuanet.com/politics/2015-11/09/c_128406178.htm (accessed Apr. 30, 2025); Liang Qiansheng, *Sisters in Guangxi both Diagnosed as “Intersex,” Possessing both Male and Female Genitalia*, Contemporary  Life news, Jan. 3, 2014, https://www.chinanews.com.cn/df/2014/01-03/5692237.shtml (accessed Apr. 30, 2025); Wang Meng, *11-Year-Old Intersex Child with both Genital Characteristics becomes Good Girl after Surgery*, Northeast  News  Network, May 8, 2006, https://news.sohu.com/20060508/n243136614.shtml (accessed Apr. 30, 2025); Ruikang Hospital Affiliated to Guangxi University of Chinese Medicine, *Intersex Abandoned Infant Undergoes Corrective Surgery to Attain Female Identity*, https://www.gxrkyy.com/news/newsyyxw/10015.html (accessed Apr. 30, 2025).

44 Wang, *supra* note 43; Ruikang Hospital Affiliated to Guangxi University of Chinese Medicine, *supra* note 43.

45 UNDP, *supra* note 12; CommonLanguage, *supra* note 12; International Labour Organization, *Inclusion of Lesbian, Gay, Bisexual, Transgender, Intersex and Queer (LGBTIQ+) Persons in the World of Work: A Learning Guide*, https://www.ilo.org/publications/inclusion-lesbian-gay-bisexual-transgender-intersex-and-queer-lgbtiq (accessed Apr. 30, 2025); Liu, *supra* note 7.

46 For instance, in a landmark case in China in 2010 (considered the first case of genetic discrimination in employment), candidates were required by employers to undergo medical examinations and submit their examination reports as part of the application process. However, those reports revealed that some candidates carried the thalassemia gene, leading to their rejection for the positions, even though the condition caused no clinical symptoms and had no impact on their ability to perform the job. See Li Jie, *Exploring Social Equity in the First Case of Genetic Discrimination*, https://zqb.cyol.com/content/2010-01/13/content_3036671.htm (accessed Apr. 30, 2025); Workers’ Daily, *Health examination reports and certificates can be obtained with money*, https://news.cctv.com/2021/09/27/ARTI7Q1Aj8B3AnpMFivOIa1P210927.shtml (accessed Apr. 30, 2025); Workers’ Daily, *Who Should Pay for Pre-Employment Medical Examinations? Experts Provide Answers*, https://www.peopleapp.com/column/30035342156-500000394828 (accessed Apr. 30, 2025).

47 UNDP & China Women’s University, *supra* note 28; Zhang & Gao, *supra* note 28.

48
*Id*.

49 PKU Law, *Analysis of the Relationship Between Labor Dispute Arbitration and Judicial Proceedings*, PKULaw, Mar. 5, 2025, https://labor.pkulaw.cn/docbook/hotfocus/3708955935428608.html (accessed Mar. 13, 2026).

50 Renmin University of China, *Renmin University—Yale Program Lecture: Constitutional Protection of the Rights of Gender-Nonconforming People in China Successfully Held*, http://humanrights.ruc.edu.cn/rqdt/gg/c8e75436c9f04272bb1c7a18780d0e72.htm (accessed Apr. 30, 2025).

51 Chengdu Business Daily, *Female Employee Dismissed for Liking to Wear Men’s Clothing, Company Ordered to Pay Compensation for Mental Distress*, Labour  Law  Website, Aug. 1, 2017, http://app.laodongfa.com/Wap/Index/show/catid/1/id/65225.html (accessed Mar. 13, 2026).

52 Zhen Xiaolv, *China’s Courts Rule for the First Time: Workplace Discrimination against Gender Expression Prohibited!,*  Town  Lawyer, Feb. 5, 2018, https://commonlanguage.github.io/TYarchives2018/20180205_1%E4%B8%AD%E5%9B%BD%E6%B3%95%E9%99%A2%E9%A6%96%E8%A1%A8%E6%80%81%EF%BC%9A%E5%8D%95%E4%BD%8D%E7%9C%8B%E4%B8%8D%E6%83%AF%E5%91%98%E5%B7%A5%E6%80%A7%E5%88%AB%E8%A1%A8%E8%BE%BE%EF%BC%9F%E6%AD%A7%E8%A7%86%EF%BC%81.html (accessed Apr. 30, 2025).

53 Liu, *supra* note 7.

54 Liu, *supra* note 7; China Labour Bulletin, *Equal Employment Rights: Workplace Resistance of China’s Sexual Minority Groups*, https://clb.org.hk/zh-hans/content/%E5%B9%B3%E7%AD%89%E5%B0%B1%E4%B8%9A%E6%9D%83%EF%BC%9A%E4%B8%AD%E5%9B%BD%E6%80%A7%E5%B0%91%E6%95%B0%E7%BE%A4%E4%BD%93%E7%9A%84%E8%81%8C%E5%9C%BA%E5%8F%8D%E6%8A%97 (accessed Apr. 30, 2025).

55 Surya Monro et al., *Intersex Equality, Diversity and Inclusion and Social Policy: Silences, Absences, and Erasures in Ireland and the UK*, 44 Crit. Soc. Policy 3–22 (2023); Council of Europe, *Intersectionality and Multiple Discrimination*, https://www.coe.int/en/web/gender-matters/intersectionality-and-multiple-discrimination (accessed Apr. 30, 2025).

56 Lu, *supra* note 15; Hu, *supra* note 15; Cao Yan-Yan et al., *Clinical, Genetic and Pathological Analysis of 165 Children with Disorders of Sex Development*, 25 Chin. J. Contemp. Pediatr. 1124–1130 (2023); Liang Yan et al., *Analysis of Gender Assignment in Children with 46, XY Disorders of Sex Development*, 41 J. Shanghai  Jiao  Tong  Univ. (Med. Sci.) 1412–1416 (2021); Yang Hongrong et al., *Discussion on the Experience of Ethical Review in the Diagnosis and Treatment of Children with Disorders of Sex Development*, 34 Chin. Med. Ethics 227–231 (2021); Chen Guangjie et al., *Progress on Evaluation, Diagnosis, and Management of Disorders of Sex Development*, 48 J. Zhejiang  Univ. (Med. Sci.) 358–366 (2019).

57 Lu, *supra* note 15; Hu, *supra* note 15; Liang et al., *supra* note 56; Group of Urological Surgery, Branch of Pediatric Surgery, Chinese Medical Association, *supra* note 26.

58 Lu, *supra* note 15; Li, *supra* note 15; Chen et al., *supra* note 56; Guo S. & Li P., *Re-Evaluation of Assigned Gender in Children with Disorders of Sex Development*, 32 Chin. J. Applied  Clin. Pediatr. 1526–1529 (2017); Yu J. et al., *Clinical Characteristics of Patients with Disorders of Sex Development after Urethroplasty*, 50 Chin. J. Clin. Physicians 1201–1206 (2020).

59 Chen R & Ren Y, *A Philosophical Examination of Children’s Autonomy Rights to Life in Informed Consent*, 43(13) Med. Philos. 31–35 (2022); Hu Xin-yue & Zhang H., *Legal Dilemma and Alleviation Measures Regarding the consent Rights of Minors in Medical Care*, 44(15) Med. Philos. 56–61 (2023); Jin Pingyue & Zhang Xinqing, *Family Refusal of Emergency Medical Treatment in China: An Investigation from Legal, Empirical, and Ethical Perspectives*, 34 Bioethics 306–317 (2020).

60 Li C. & Tian Y., *Issues and Approaches of Informed Consent in Non-Therapeutic Medical Trials for Children*, 6 Ethics  Research 118–123 (2017).

61 Reforms in medically assisted reproduction and other urgent regulations, FEK A’ 142/21.7.2022. N. 4958/2022.

62 Leġiżlazzjoni Malta, *supra* note 11.

63 Law 4/2023, B.O.E. No. 51, Feb. 28, 2023 (Spain), https://www.boe.es/eli/es/l/2023/02/28/4 (accessed Apr. 30, 2025).

64 Although Chinese law requires parents (or guardians) to act in the best interests of the child, this principle is generally stated in broad terms and lacks specific guidance on how it should be operationalized in clinical decision-making, particularly in cases involving irreversible medical interventions on intersex children. Although there is growing attention in China to the issue of children’s autonomy in medical decision-making, in practice, such decisions are typically made by parents in consultation with doctors, without institutional mechanisms for obtaining the child’s assent or for deferring non-urgent procedures until the child is capable of meaningful participation. See Chen & Ren, *supra* note 59; Hu & Zhang, *supra* note 59; Li & Tian, *supra* note 60.

65 According to Law of the P.R.C. on the Protection of Minors, art. 16, the parents or other guardians of minors are required to fulfill their guardianship duties and serve as legal representatives for minors in civil matters.

66 See Lu, *supra* note 15; Xiao, *supra* note 27.

67 Yang et al., *supra* note 56; Hu, *supra* note 15; Lu, *supra* note 15; Guo & Li, *supra* note 58.

68 CommonLanguage, *supra* note 12; Lu, *supra* note 15; Li, *supra* note 15.

69 Melanie Newbould, *When Parents Choose Gender: Intersex, Children, and the Law*, 24 Med. Law  Rev. 474–496 (2016); Frederique Joosten, *Reaffirming Autonomy of Trans and Gender-Diverse Children and Adolescents*, 37 Int. J. Law  Policy  Fam. 1–4 (2023); Edmund M. Horowicz, *Intersex Children: Who are We Really Treating?*, 17 Med. Law  Int. 183–218 (2017).

70 Ye, *supra* note 25; Cao et al. *supra* note 56; Chinese Medical News, *Consensus on Pediatric Endocrine Diagnosis and Treatment of Disorders of Sex Development Released*, https://www.cn-healthcare.com/api/third/article/522396 (accessed Apr. 30, 2025).

71 Chen et al., *supra* note 56; Yang et al., *supra* note 56; Yu et al., *supra* note 58; Cao et al., *supra* note 56; Guo Qiang et al., *Diagnosis and Surgical Treatment of 46, XY Disorders of Sex Development*, 41 J. Int. Reprod. Health/Fam. Plan. 30–36 (2022).

72 Lu, *supra* note 15; Hu, *supra* note 15; Xiao, *supra* note 27.

73 Chen et al., *supra* note 56; Liang et al., *supra* note 56; Yu et al., *supra* note 58; Yang et al., *supra* note 56; Group of Urological Surgery, Branch of Pediatric Surgery, Chinese Medical Association, *supra* note 26.

74 Li, *supra* note 15; Silvia M. Cuadra et al., *Normalizing Intersex Children through Genital Surgery: The Medical Perspective and the Experience Reported by Intersex Adults*, 27 Sexualities 1–20 (2022); Morgan Carpenter, *The “Normalization” of Intersex Bodies and “Othering” of Intersex Identities in Australia*, 15 J. Bioeth. Inq. 487–495 (2018).

75
*Id.*

76 Lu, *supra* note 15; Li, *supra* note 15; Xiao, *supra* note 27.

77
*Id.*

78 Kate Hodkinson, *The Need to Know—Therapeutic Privilege: A Way Forward*, 21 Health  Care  Anal. 105–129 (2013); Claude Richard et al., *Therapeutic Privilege: Between the Ethics of Lying and the Practice of Truth*, 36 J. Med. Ethics 353–357 (2010).

79 For example, Article 61 of the Implementation Rules of the Regulations on the Administration of Medical Institutions states: “Medical institutions shall implement protective medical measures during diagnosis and treatment activities and obtain the cooperation of the patient’s family and relevant parties.” Article 62 further specifies: “Medical institutions shall respect the patient’s right to be informed about their condition, diagnosis, and treatment. When performing surgery, special examinations, or special treatments, necessary explanations should be provided to the patient. If it is not appropriate to inform the patient due to protective medical measures, relevant information should be communicated to the patient’s family.” Many medical institutions have also issued rules stating that doctors and nurses should take care to implement protective medical measures. During rounds or while conducting various examinations and treatments, they are advised to refrain from discussing or explaining content that may affect patients emotions or increase their mental burden in front of them. See China Rehabilitation Research Center, *Protective Medical System*, https://www.crrc.com.cn/Html/News/Articles/9673.html (accessed Apr. 30, 2025).

80 Liu J., Obligation, *Informed Consent, and Health-Care Reforms in China*, 11 Asian  Journal of  Law and  Society 343 (2024); Jingru Li, *Family-Oriented Informed Consent in China’s Clinical Settings: A Sociological and Ethical Study (Doctoral Dissertation, University of Otago)*, University of  Otago  Research  Portal, https://ourarchive.otago.ac.nz/esploro/outputs/doctoral/Family-oriented-informed-consent-in-Chinas-clinical/9926479521601891 (accessed Mar. 13, 2026).

81 Liang Zhanming et al., *Patient-Centred Care and Patient Autonomy: Doctors’ views in Chinese hospitals*, 23 BMC Med. Ethics 1–12 (2022).

82 See Chen et al., *supra* note 56; Cao et al., *supra* note 56; Chinese Medical News, *supra* note 70; Yang et al., *supra* note 56; Liang et al., *supra* note 56; Group of Urological Surgery, Branch of Pediatric Surgery, Chinese Medical Association, *supra* note 26.

83 See Liang et al., *supra* note 56; Yu et al., *supra* note 58; Yang et al., *supra* note 56; Group of Urological Surgery, Branch of Pediatric Surgery, Chinese Medical Association, *supra* note 26.

84 See Ye, *supra* note 25; Chen et al., *supra* note 56; Liang et al., *supra* note 56; Cao et al., *supra* note 56; Yang et al., *supra* note 56; Xiao, *supra* note 27.

85 See Yang et al., *supra* note 56; Liao Kang & Chen Jie, *A Phenomenological Study on Psychological Experience of Patients with True Hermaphroditism During Treatment*, 18 Mod. Clin. Nurs. 32–36 (2019).

86 See National Health Commission of China, *G05 Rules on Gender Reassignment Surgery Clinical Application Management*, https://cnlgbtdata.com/files/uploads/2022/08/%E6%80%A7%E5%88%AB%E9%87%8D%E7%BD%AE%E6%8A%80%E6%9C%AF%E4%B8%B4%E5%BA%8A%E5%BA%94%E7%94%A8%E7%AE%A1%E7%90%86%E8%A7%84%E8%8C%832022%E5%B9%B4%E7%89%88.pdf (accessed Apr. 30, 2025).

87 In China, the public security authorities are responsible for managing household registration and issuing official identity documents. Applications to update gender markers are also processed by local public security bureaus. This administrative procedure for gender marker change was confirmed by the Ministry of Public Security in 2002 and 2008, in response to inquiries from provincial public security bureaus regarding specific cases. The Ministry of Public Security clarified that applicants must submit proof of gender-affirming surgery from a government-designated hospital, along with a gender identification certificate from a certified institution. A more formalized procedure was subsequently established in 2011, which will be discussed in detail later in the main text. See Ministry of Public Security of China, *Catalogue of Current Effective Rules and Normative Documents of the Ministry of Public Security*, https://www.gov.cn/gongbao/content/2011/content_1913192.htm (accessed Apr. 30, 2025).

88 See Xianfa (1982) (China) https://www.gov.cn/guoqing/2018-03/22/content_5276318.htm (accessed Apr. 30, 2025).

89 For example, Articles 33 and 34 of the Constitution of the People’s Republic of China stipulate that all citizens are equal before the law, and the state respects and guarantees human rights. All citizens enjoy equal rights, including the right to vote and be elected, without discrimination based on ethnicity, race, gender, occupation, family background, religious belief, education level, property status, or length of residence, except those deprived of political rights by law.

90 See Xianfa art. 38 (1982) (China).

91 Lin Xiaowen, *An Empirical Study on the Citation of Constitutional Norms in Judicial Decisions in China*, https://www.legal-theory.org/?act=view&id=21989&mod=info (accessed Apr. 30, 2025); Guo Xiaoyan & Wei Caili, *Empirical Study of Judicial Judgments Invoking the Constitutional Argument*, 1 Yangtze  Tribune 84–90 (2021); Feng Jianpeng, *Constitutional Reference and its Functions in Judicial Decisions in China*, 39 Chin. J. Law 44–59 (2017).

92
*Id*.

93 Civil Code of the People’s Republic of China, (2020), National People’s Congress of the Republic of China, Available at: https://www.gov.cn/xinwen/2020-06/01/content_5516649.htm (accessed Apr. 30, 2025).

94 Civil Code of the People’s Republic of China, art. 4, 14, (2020).

95 Civil Code of the People’s Republic of China, art. 1004, (2020).

96 See Chen & Ren, *supra* note 59; Hu & Zhang, *supra* note 59; Li & Tian, *supra* note 60.

97 The principle of the best interests of the child is affirmed in Article 4 of the Law on the Protection of Minors of China and Article 35 of China’s Civil Code. It is also reflected in international human rights law, including the Convention on the Rights of the Child, which establishes the best interests of the child as a primary consideration in all actions concerning children.

98 In this regard, parental consent on medical decisions alone may not guarantee alignment with the child’s best interests. See Hu & Zhang, *supra* note 59; Legal Daily, *Experts Suggest Revoking Parental Custody Rights when Parents Prevent Sick Children from Receiving Medical Treatment*, http://legalinfo.moj.gov.cn/zhfxfzzx/fzzxyw/202304/t20230406_475757.html (accessed Apr. 30, 2025).

99 See Cuadra et al., *supra* note 74; Carpenter, *supra* note 74; Human Rights Watch, *supra* note 10.

100 See Cuadra et al., *supra* note 74; Lu, *supra* note 15; Li, *supra* note 15; Xiao, *supra* note 27.

101 Civil Code of the People’s Republic of China, art. 110, 990, 1003, (2020).

102 Zhang Bei, *Professor Li Yan’s New Work: An Interpretation of the Civil Code on Models of Medical Decision-Making for Minors*, Civil and  Commercial  Law  School of  Shandong  University of  Political  Science and  Law, Jun. 21, 2021, https://cclc.sdupsl.edu.cn/info/1005/1434.htm (accessed Mar. 13, 2026).

103 Civil Code of the People’s Republic of China, art. 1219, (2020).

104 Civil Code of the People’s Republic of China, art. 27, 34, (2020).

105 Civil Code of the People’s Republic of China, art. 35, (2020).

106 See Lu, *supra* note 15; Li, *supra* note 15.

107 See Human Rights Watch, *supra* note 10; Cuadra et al., *supra* note 74; Morgan Carpenter, *The Human Rights of Intersex People: Addressing Harmful Practices and Rhetoric of Change*, 24 Reprod. Health  Matters 74–84 (2016); The Office of the United Nations High Commissioner for Human Rights, *OHCHR Technical Note on the Human Rights of Intersex People: Human Rights Standards and Good Practices*, https://www.ohchr.org/en/documents/tools-and-resources/ohchr-technical-note-human-rights-intersex-people-human-rights (accessed Apr. 30, 2025).

108 Civil Code of the People’s Republic of China, Part 7, Chapter 6, (2020).

109 Civil Code of the People’s Republic of China, art. 1219, (2020).

110 According to Article 3 of the Law on Resident Identity Cards of China, the items registered on the resident identity card include: name, gender, ethnicity, date of birth, address of permanent residence, citizen identification number, photograph, fingerprint information, validity period of the card, and issuing authority. The citizen identification number is a unique, lifelong identity code assigned to each citizen by the public security organs in accordance with the national standard for citizen identification numbers.

111
*Id*.

112 The State Bureau of Quality and Technical Supervision of China, *National Standard for Citizen Identification Numbers (GB 11643–1999)*, https://openstd.samr.gov.cn/bzgk/gb/newGbInfo?hcno=080D6FBF2BB468F9007657F26D60013E (accessed Apr. 30, 2025).

113 See National Health Commission of China, *supra* note 86.

114 See Ministry of Public Security of China, *supra* note 87.

115 Ministry of Public Security of China, *Notice on the Issuance of the Rules for Household Registration and Resident Identity Card Management (Trial)*, https://www.beijing.gov.cn/gate/big5/www.beijing.gov.cn/zhengce/zhengcefagui/202211/t20221116_2860246.html (accessed Apr. 30, 2025).

116 This verification step is part of the duties assigned to local public security bureaus under China’s household registration framework. See *Id*.

117 For example, according to Article 3 of the Labor Law of China (2018 Amendment), workers have the right to equal employment and choice of occupation, equal access to wages, rest and leave, labor safety and health protection, vocational training, social insurance and welfare benefits, the right to request the resolution of labor disputes, and other labor rights as prescribed by law. Additionally, Article 26 of the Employment Promotion Law of China (2015 Amendment) and Article 9 of the Regulations on Employment Services and Employment Management (2022 Amendment) state that employers and employment agencies must provide equal employment opportunities and fair employment conditions to workers, and must not engage in employment discrimination.

118 See Labor Law of China, art. 12, (2018 Amendment); Employment Promotion Law of China, art. 3, (2015 Amendment); Regulations on Employment Services and Employment Management, art. 4, (2022 Amendment).

119 Moreover, as mentioned earlier, although the court in that case acknowledged that gender identity and expression fall within the scope of general personality rights and should not be subject to discrimination, China’s legal system does not follow a precedent-based model. This ruling does not necessarily carry binding authority in future cases.

120 Labor Law of China, art. 13, (2018 Amendment); Employment Promotion Law of China, art. 27, (2015 Amendment); Regulations on Employment Services and Employment Management, art. 16, (2022 Amendment); Law on Safeguarding the Rights and Interests of Women, art. 43, (2022 Amendment).

121 Employment Promotion Law of China, art. 27, (2015 Amendment), Regulations on Employment Services and Employment Management, art. 16, (2022 Amendment), Law on Safeguarding the Rights and Interests of Women, art. 44, (2022 Amendment).

122 Law on Safeguarding the Rights and Interests of Women, art. 25, (2022 Amendment).

123 See Law on Resident Identity Cards of China, *supra* note 110; The State Bureau of Quality and Technical Supervision of China, *supra* note 112.

124 See Small Luk, *supra* note 7; Liu, *supra* note 7; Lu, *supra* note 15; Xiao, *supra* note 27.

125 See Small Luk, *supra* note 7; Liu, *supra* note 7; Liu Renwen and Gao Yu, *Reflections on the De-Gendering of Criminal Law*, People’s  Court  Daily, Aug. 10, 2017, https://gqcfy.jxfy.gov.cn/article/detail/2017/08/id/4962982.shtml (accessed Mar. 13, 2026).

126 Qiu D. et al., *Public Mental Health Services in Southern China and Related Health Outcomes among Individuals Living with Severe Mental Illness*, 9 Global  Health  Research and  Policy 31 (2024); Xiang Y. et al., *Rethinking Progress and Challenges of Mental Health Care in China*, 17(2) World  Psychiatry 231–232 (2018).

127 UN Committee on the Rights of the Child, *Concluding Observations on the Combined Fourth and Fifth Periodic Reports of Chile*, https://docs.un.org/en/CRC/C/CHL/CO/4-5 (accessed Apr. 30, 2025); UN Committee on the Rights of the Child, *Concluding Observations on the Combined Third to Fifth Periodic Reports of Nepal*, https://docs.un.org/en/CRC/C/NPL/CO/3-5 (accessed Apr. 30, 2025); UN Independent Expert on Protection Against Violence and Discrimination Based on Sexual Orientation and Gender Identity, *Protection Against Violence and Discrimination Based on Sexual Orientation and Gender Identity*, https://docs.un.org/en/A/73/152 (accessed Apr. 30, 2025).

128 This evolving consensus is also evidenced by many UN bodies, such as the UN Human Rights Committee, the UN Committee Against Torture, and the UN Committee on the Rights of the Child. They also call for informed consent to be ensured and for access to redress through compensation and accountability mechanisms. See Universal Human Rights Index, *View Document Kenya: Concluding Observations*, https://uhri.ohchr.org/en/document/f2995a60-87ba-4b50-ba0f-76037e716d40 (accessed Apr. 30, 2025); Universal Human Rights Index, *View Document Finland: Concluding Observations*, https://uhri.ohchr.org/en/document/cb63d72a-d1f0-42b6-9aad-d7fa015c316a (accessed Apr. 30, 2025); UN Committee Against Torture, *Concluding Observations on the Seventh Periodic Report of France*, https://documents.un.org/doc/undoc/gen/g16/117/35/pdf/g1611735.pdf?OpenElement (accessed Apr. 30, 2025); Universal Human Rights Index, *View Document CRC/C/GBR/CO/5*, https://uhri.ohchr.org/en/document/b6cf22f9-7755-4865-8fa7-125173502636 (accessed Apr. 30, 2025).

129 Universal Human Rights Index, *View Document Switzerland: Concluding Observations*, https://uhri.ohchr.org/en/document/010ef405-2f41-4427-a39b-78f31a850294 (accessed Apr. 30, 2025); The Office of the United Nations High Commissioner for Human Rights, *Born Free & Equal: Sexual Orientation, Gender Identity and Sex Characteristics in International Human Rights Law (Second Edition)*, https://www.ohchr.org/sites/default/files/Documents/Publications/Born_Free_and_Equal_WEB.pdf (accessed Apr. 30, 2025).

130 UN Human Rights Committee, *Concluding Observations on the Sixth Periodic Report of Mexico*, https://docs.un.org/en/CCPR/C/MEX/CO/6 (accessed Apr. 30, 2025); UN Human Rights Committee, *Concluding observations on the sixth periodic report of Uruguay*, https://tbinternet.ohchr.org/_layouts/15/treatybodyexternal/Download.aspx?symbolno=CCPR%2FC%2FURY%2FCO%2F6&Lang=en (accessed Apr. 30, 2025).

131 World Health Organization, *Sexual Health, Human Rights and the Law*, https://www.who.int/publications/i/item/9789241564984 (accessed Apr. 30, 2025); World Medical Association, *WMA Statement on Forced or Coerced Sterilization*, https://www.wma.net/policies-post/wma-statement-on-forced-and-coerced-sterilisation/ (accessed Apr. 30, 2025); International Labour Organization, *supra* note 44.

132 See Leġiżlazzjoni Malta, *supra* note 11.

133 Lei n.° 38/2018, de 7 de agosto [Act no. 38/2018 of 7 August], https://diariodarepublica.pt/dr/detalhe/lei/38-2018-115933863 (accessed Apr. 30, 2025) (Port.); Argentina Ministerio de Justicia de la Nación, 23/5/2012, “Establécese el derecho a la identidad de género de las personas,” La Ley [L.L.] (Ley 2012–26-743) (Arg.), http://servicios.infoleg.gob.ar/infolegInternet/anexos/195000-199999/197860/norma.htm (accessed Apr. 30, 2025).

134 See Australia’s Federal Register of Legislation, *supra* note 11.

135 The Fair Work Ombudsman of the Australian Government, *Gender Equality Measures and Small Claims Process*, https://www.fairwork.gov.au/about-us/workplace-laws/legislation-changes/secure-jobs-better-pay/gender-equality-measures-and-small-claims-process (accessed Apr. 30, 2025).

136 ACT Government, *Protecting the Rights of People with Variations in Sex Characteristics*, https://www.act.gov.au/health/topics/lgbtiqa-health/protecting-the-rights-of-people-with-variations-in-sex-characteristics (accessed Apr. 30, 2025).

137 FEK A’ 142/21.7.2022. N. 4958/2022, *supra* note 61; Law 4/2023, B.O.E. No. 51, Feb. 28, 2023 (Spain), *supra* note 63.

138 Limor M. Danon et al., *Opportunities and Challenges with the German Act for the Protection of Children with Variations of Sex Development*, 35 Int. J. Impot. Res. 38–45 (2023).

139 International Commission of Jurists, *In re Völling, Regional Court Cologne, Germany (6 February 2008),*  https://www.icj.org/sogicasebook/in-re-volling-regional-court-cologne-germany-6-february-2008/ (accessed Apr. 30, 2025); Susanne Baller, *Michaela Raab klagt gegen “Zwangstranssexualisierung”*, http://www.stern.de/familie/leben/operation-an-intersexuellem-menschen-michaela-raab-klagt-gegen-zwangstranssexualisierung-2176273.html (accessed Apr. 30, 2025).

140 See Government of the Hong Kong Special Administrative Region, *supra* note 14.

141 China ratified the United Nations Convention on the Rights of the Child in 1992 and is legally bound to uphold its principles, including the best interests of the child. See UNICEF China, *China Marks 20th Anniversary of its Ratification of the Convention on the Rights of the Child*, https://www.unicef.cn/en/press-releases/china-marks-20th-anniversary-its-ratification-convention-rights-child (accessed Apr. 30, 2025).

142 Ministry of Foreign Affairs of the People’s Republic of China, *Combined Fifth and Sixth Periodic Reports of the People’s Republic of China on the Implementation of the Convention on the Rights of the Child*, https://www.mfa.gov.cn/web/wjb_673085/zfxxgk_674865/gknrlb/tywj/tyqk/202311/t20231122_11185101.shtml (accessed Apr. 30, 2025).

143 See The Office of the United Nations High Commissioner for Human Rights, *supra* note 107.

144 For instance, Article 19 of the Civil Code recognizes that minors over the age of eight possess limited civil capacity and may independently carry out civil legal acts that are appropriate to their age and understanding. In addition, Article 18 allows minors aged 16 and above to be deemed fully capable if their main source of income is self-support. While these provisions do not directly govern medical consent, they offer a reference point for defining the developmental maturity required for meaningful participation in medical decision-making.

145 Wu Y. et al., *The Current State and Challenges of Clinical Ethics Consultation for Prenatal Diagnosis: A Qualitative Study of Committee Employee Perspectives in China*, 17 Asian  Bioethics  Review 73 (2024); Xie Guangkuan, *Clinical Ethics Consultation and Education Seminar Successfully Held at Peking University Health Science Center*, School of  Health  Humanities, Peking  University, May 22, 2024, https://shh.bjmu.edu.cn/ybxw/0fb673cef91d4bcb9f4569270029e953.htm (accessed Mar. 13, 2026).

146
*Id*.

147 Xie Z. & Gong X., *Review on the Operation Mechanism of Hospital Ethics Committees in China*, 34 Chinese  Medical  Ethics 433 (2021); Ministry of Science and Technology of the People’s Republic of China, *Promoting Responsible Science and Steer Ethics Well: Official from the Ministry of Science and Technology Interprets the Measures for the Review of Science and Technology Ethics (Trial)*, Ministry of  Science and  Technology of the  People’s  Republic of  China, Oct. 9, 2023, https://www.most.gov.cn/xxgk/xinxifenlei/fdzdgknr/fgzc/zcjd/202310/t20231010_188399.html (accessed Mar. 13, 2026).

148 See Hu & Zhang, *supra* note 59.

149 In China, judicial interpretations are authoritative normative documents issued by the Supreme People’s Court or the Supreme People’s Procuratorate to clarify the application of laws in specific contexts. While not formally part of the legislative hierarchy, judicial interpretations play a crucial role in guiding courts across the country and are widely regarded as authoritative sources of legal interpretation with quasi-legislative effect. These interpretations are often issued in response to widespread legal controversies or inconsistent judgments across the country. By clarifying legislative intent and harmonizing judicial reasoning, they help ensure more uniform application of the law nationwide. See Xu J., *How Judicial Interpretations are Made: A Focus*, People’s  Daily  Online, 2015, http://theory.people.com.cn/n/2015/0114/c40531-26381594.html (accessed Apr. 30, 2025); Xu Ridan, *Supreme People’s Procuratorate: The Key to Formulating Judicial Interpretations Lies in Solving the Real Problems and Urgent Needs of Frontline Judicial Case Handling*, Supreme  People’s  Procuratorate of the  People’s  Republic of  China, Feb. 24, 2025, https://www.spp.gov.cn/spp/2025zgjtzwlftzb/202502/t20250224_685237.shtml (accessed Apr. 30, 2025).

150 See Universal Human Rights Index, *supra* note 129.

151 Australian Human Rights Commission*, Ensuring Health and Bodily Integrity*, https://humanrights.gov.au/intersex-report-2021 (accessed Apr. 30, 2025).

152 See The Office of the United Nations High Commissioner for Human Rights, *supra* note 107.

153 See Knoppers et al., *supra* note 9.

154 Egale Canada, *Intersex Community Hub*, https://egale.ca/awareness/intersexhub/ (accessed Apr. 30, 2025); Intersex Canada, *Support & Resources*, https://intersexcanada.org/support-resources/ (accessed Apr. 30, 2025); Centre of Genomics and Policy, *Toward Affirmative Intersex Health Communication in Canada: An Introductory Guide for Healthcare Providers*, https://www.genomicsandpolicy.org/Ressources/36.pdf (accessed Mar. 25, 2026).

155 See PrideLab, *supra* note 14; Family Planning Association of Hong Kong, *supra* note 14; Beyond the Boundary-Knowing and Concerns Intersex, *LC Paper No. CB(2)1266/17–18(07),*  https://www.legco.gov.hk/yr17-18/chinese/panels/ca/papers/ca20180430cb2-1266-7-ec.pdf (accessed Apr. 30, 2025).

156 Intersex Asia, *Asian intersex Statement 2023*, https://intersexasia.org/wp-content/uploads/2024/09/Asian_statement_2023_Official_27Dec.pdf (accessed Apr. 30, 2025).

